# Linking geography, isolation source, and genomic diversity in a global *Candida albicans* phylogeny

**DOI:** 10.1038/s44512-026-00003-2

**Published:** 2026-11-05

**Authors:** Abdul-Rahman Adamu Bukari, Dana A. Opulente, Chris Todd Hittinger, Christophe d’Enfert, Christina A. Cuomo, Aleeza. C. Gerstein

**Affiliations:** 1 Department of Microbiology, University of Manitoba, Winnipeg, MB Canada; 2 Laboratory of Genetics, DOE Great Lakes Bioenergy Research Center, Center for Genomic Science Innovation, J. F. Crow Institute for the Study of Evolution, Wisconsin Energy Institute, University of Wisconsin-Madison, Madison, WI USA; 3 Institut Pasteur, Université Paris Cité, INRAE USC2019, Unité Biologie et Pathogénicité Fongiques, Paris, France; 4 Brown University Division of Biology and Medicine, Providence, RI USA; 5 Broad Institute, Cambridge, MA USA; 6 Statistics Department, University of Manitoba, Winnipeg, MB Canada; 7 Present Address: Biology Department, Villanova University, Villanova, PA USA

**Keywords:** Computational biology and bioinformatics, Evolution, Genetics, Microbiology

## Abstract

*Candida albicans* is a common commensal species in the human microbiome at multiple sites and an opportunistic pathogen. Previous phylogenomic analyses identified major clades but often relied on imprecise genomic methods, limited geographic and ecological sampling, and non-standardized strategies for phylogenetic resolution. Here, we address these gaps by reconstructing a whole-genome phylogeny to examine how geography and site of isolation contribute to phylogenetic structure in *C. albicans*. We analyzed phased genomes from 945 global isolates obtained across diverse clinical and ecological contexts and applied an agnostic, threshold-based clustering approach to systematically define cluster boundaries and quantify intraspecific genomic features. Our analyses preserved previously defined major clusters while identifying six novel clusters, predominantly composed of highly admixed Asian isolates. Although geographic origin and isolation source were each significantly associated with cluster membership, these associations were confounded because isolates from specific regions were disproportionately derived from particular sources. However, an analysis of environmental isolates alongside a diverse strain collection from Winnipeg, Canada, highlights the potential for frequent local transmission. More than 95% of isolates were heterozygous at the mating-type locus, while aneuploidy was present in 8% of isolates distributed across the phylogeny. Analysis of the *AGO1* PAZ domain involved in RNAi interference identified both known and novel RNAi variants, predominantly in a heterozygous state. Intra-host analysis of isolates from 95 individuals revealed predominantly clonal colonization, though fourteen individuals harbored isolates from different clusters. Together, these findings refine the phylogenetic structure of *C. albicans* and provide insights into genomic plasticity and strain transmission in this common fungal species.

## Introduction

*Candida albicans* is a common opportunistic human fungal pathogen responsible for a wide range of conditions, from superficial to life-threatening^1–3^. *C. albicans* is also commonly found as a commensal species in many of the same body sites where it causes disease^4–6^. The switch to pathogenicity is known to occur when the local microbiota is disrupted, tissue barriers are compromised, or immune defences are weakened; thus, the same strains that exist as commensals are often implicated in disease. *C. albicans* has also been isolated from animals that are closely associated with humans^7^, as well as human-associated environments^8^. Environmental isolates of *C. albicans* have only been rarely studied, with fewer than 20 isolates from sources such as sand, soil, tree bark, duff, and fruit reported to date^9–11^. Most have not yet been included in phylogenetic studies, though two out of three *C. albicans* isolates sequenced from oak tree bark were closely related to human isolates, while the third was not assigned to an existing cluster^10^. The transmission dynamics of *C. albicans* are not fully resolved. Humans are typically colonized at birth by their mother^12,13^, and available evidence suggests that commensal strains generally remain stable within individuals in early life^14^. However, detailed longitudinal data on within-host strain pesistence, diversity, and microevolution remain limited^15^.

*C. albicans* is typically diploid; its genome encodes ~6000 genes with a haploid genome size of about 14.5 Mb^16^. *C. albicans* is predominantly asexual and hence its genome is at least partially released from the karyotypic constraint imposed by frequent meiosis. In addition to “standard” single-nucleotide variants (SNVs) and short insertions and deletions (INDELS), short- and long-tract copy-number changes, loss of heterozygosity (LOH), and chromosomal aneuploidy are also frequently observed in lab experiments and among clinical isolates. It is often hypothesized that aneuploidy, which occurs at a higher mutation rate than the per-base pair mutation rate, enhances the ability of *C. albicans* to persist and thrive in the wide range of environments it encounters in the human body^17,18^. Epigenetic variation, which enables rapid acclimation, may also play a role in the wide niche breadth. RNAi was long thought to be inactive in *C. albicans* because the reference strain, SC5314, carries a homozygous inactivating missense mutation in the PAZ domain of Ago1 (Argonaute), the central RNAi component. However, a recent assessment of 295 additional isolates found that only eight isolates carry the missense variant^19^. Of these, seven variants are heterozygous and likely retain RNAi activity, while only one additional isolate was homozygous for the inactive SC5314 variant.

The 182-isolate short-read whole-genome sequencing (WGS) phylogeny widely used for *C. albicans* was published in 2018; we refer to this tree as the “existing WGS tree”^20^. The existing WGS tree comprises 17 distinct genetic clusters, including 12 numbered in parallel to the clusters (also referred to synonymously as clades) previously identified by multilocus sequence typing^21,22^. The cutoff value used for cluster designation analysis from the MLST study was itself partially based on clusters identified by an earlier DNA-fingerprinting method using the moderately repetitive Ca3 probe^23,24^. As stated in the MLST manuscript^22^, the 0.04 cutoff was somewhat arbitrary, used mainly for the convenience of comparative isolate analyses, yet the majority of subsequent phylogenetic studies using updated genome sequencing techniques have adhered to it seemingly without in-depth reevaluation^10,20,25^. The five new clusters identified in the WGS data were assigned alphabetical designations (A–E); each contained fewer than 10 isolates. Support for the existing WGS cluster delineation was found from NGSAdmix analysis (with k = 13), which identified the same clusters. NGSAdmix additionally indicated extensive admixture in the ten isolates that did not group with the clusters and were labeled as singletons, and provided evidence for recombination in common ancestors leading to new WGS cluster A (between clusters 3 and D) and cluster B (between clusters 2 and E). A recent admixture analysis of MLST data from over 5000 isolates identified extensive admixture across most geographic populations (with k = 2), consistent with frequent gene flow^26^. Since the existing WGS tree was published, new WGS studies that seek to generate a phylogeny typically start with their 182 isolates and follow their cluster designations. An exception is the recent study by Schikora-Tamarit and Gabaldón (2024), which defined clades based on support-based node selection and branch-length optimization^27^. From 642 isolates (458 natural isolates plus 184 in vitro evolved or engineered), the authors identified 21 clades: 17 corresponded to clades from the existing WGS tree, four new clades, and 10 singleton isolates^27^.

In some fungal species, isolates within the same cluster tend to have similar geographic origins, virulence, and/or resistance patterns (*e.g.*, Candidozyma auris^28^, *Saccharomyces cerevisiae*^29^, *Aspergillus fumigatus*^30,31^). The pattern in *C. albicans* seems more nuanced, though in some studies, isolates from the same geographic region cluster together. For example, recent MLST analyses of vulvovaginal isolates from northern China revealed a novel cluster of 92 *C. albicans* isolates^32^, while a study of isolates from Thailand found that ~25% (13/46) clustered together in MLST cluster 17^33^, though this cluster also contains isolates from other continents^22^. It stands to reason that any potential geographical specificity of clusters is gradually being lost, perhaps due to the high global rate of human movement, which facilitates not just transmission of isolates but also increases the possibilities of recombination events producing novel lineages^22,32,34^. *C. albicans* may be particularly susceptible to this due to its high colonization prevalence in healthy hosts compared to other fungal opportunistic pathogens such as *Cryptococcus neoformans* and *C. auris*.

Many studies have sought to determine whether there is a link between cluster and phenotypic traits of clinical interest. Cluster 1 isolates are the most likely to be resistant to flucytosine, achieved through the R101C mutation in *FUR1* that is not observed in flucytosine-resistant isolates from other clusters^35–37^. Cluster 1 isolates are also the most likely to be resistant to terbinafine compared with isolates from clusters 2, 3, 4, and 11isolates^37^, yet no cluster-associations were observed for a panel of seven other common antifungal drugs. Isolates in cluster 2 have significantly lower acid phosphatase activity than those in clusters 1 and 3^38^. Significant differences have also been identified among isolates in different clusters (1, 2, 3, 4) in relation to intergenic tandem repeat sequence alleles of gene families that encode *C. albicans* surface proteins that play a role in adhesion to host surfaces (*ALS2*, *ALS4*, *ALS6*, *ALS7*, *ALS9*, *HYR1*, and *HYR2*)^38^. Blood isolates from cluster 17 had greater hemolytic activity compared to isolates from eight other clusters, though no differences were found for proteinase activity, phospholipase activity, growth in bovine serum albumin, adherence to plastic, growth at different temperatures, or biofilm formation^38^. A significantly higher proportion of isolates from cluster 1 are associated with superficial infections and commensal carriage than isolates from other clusters, and cluster 1 is consistently the largest, regardless of the phylogenetic method used. It has been hypothesized that cluster 1 isolates may have an enhanced ability to evade host defenses due to the presence of a 985 bp *HpaII* fragment (*MU13-4*), which potentially has a role in generating genetic variability. Whether the identified associations have clinical relevance in a predictive manner that could inform treatment decisions remains an open area of study^39^.

To improve phylogenetic resolution, address the aforementioned limitations in cluster designation, and assess potential signatures of geography and sites of isolation, we reconstructed a new short-read whole-genome sequencing (WGS) phylogeny for *C. albicans*. We used data from 1178 isolates, including 1130 human-associated isolates, 31 environmental isolates, and 17 isolates of unknown origin. Among these, 85 are newly sequenced human-associated isolates from Manitoba, Canada, and seven are environmental isolates from the United States, with the remainder sourced from the NCBI SRA archive. We incorporated phased haplotype information and used a statistical threshold-based method for cluster assignment. This expanded and rigorously analyzed dataset updates our understanding of the *C. albicans* phylogenetic structure, including the geographic and site-specific distribution of genome-wide heterozygosity, aneuploidies, and RNAi-deficiencies. We also highlight how biased sampling has led to continuing knowledge gaps in the relationships among isolates between and within populations.

## Results

**Fig. 1:**
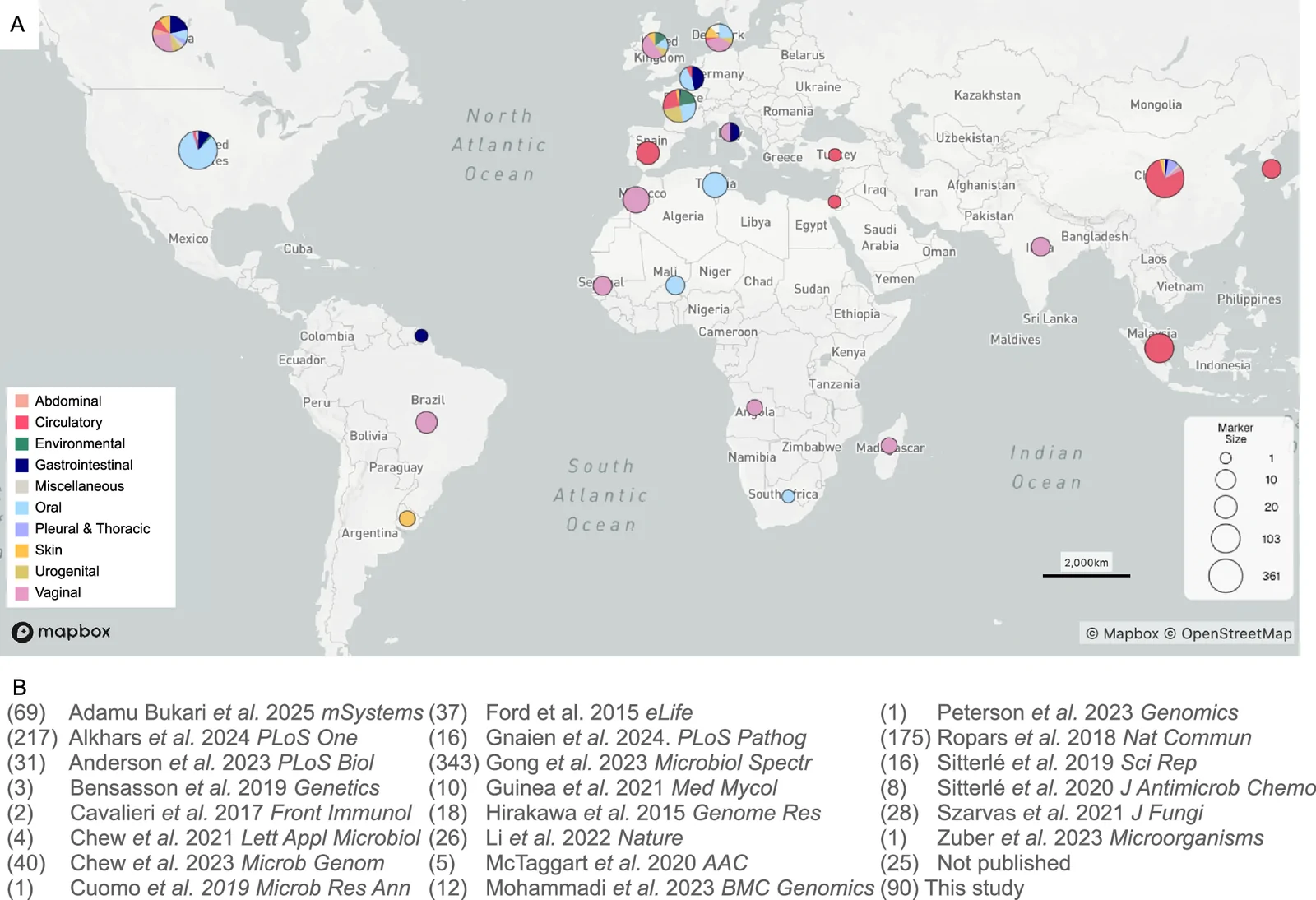
Distribution and literature source of isolates used in this study. **A** Geographical distribution of the 1178 isolates analyzed in this study. Circle size is proportional to the log of the number of isolates from each region, while colored sectors represent their respective isolation sources. This includes 37 cluster 13 (*Candida africana*) isolates. The image was generated using Microreact^92^, which renders the maps through Maps © Mapbox (www.mapbox.com/about/maps) and © OpenStreetMap (www.openstreetmap.org/about). **B** Publication source of the isolates used. The number of isolates from each manuscript is provided in brackets.

1178 *C. albicans* isolates with WGS data obtained from 26 countries across five continents were included in the study. Geographic and site-of-isolation distributions were highly uneven (Fig. 1 and Table S1). North America (520 isolates) and Asia (410 isolates) were the best-represented continents, with many fewer isolates from Europe (179), Africa (51), and South America (10); no isolates came from Australia. At the country level, sampling was even sparser; only 11 countries had at least ten isolates. Unfortunately, the majority of isolates from each country came from a single isolation source as only eight countries had isolates from multiple sources. Approximately one-third of all isolates were acquired from the bloodstream, and another third were from the oral cavity. The “environmental” isolates included 19 from food spoilage (all from France), two from birds (both from France), and ten from trees, duff, soil, or fruit (three from oak trees in the UK, with the other seven from across the United States). This uneven distribution was likely influenced by many factors, such as regional research focus and funding availability. Thus, although this study represents the largest global survey to date for *C. albicans*, there are almost certainly biologically relevant worldwide regional differences among isolates that are not all captured here.

### Topological differences in constructing phylogenies with haplotype information

**Fig. 2:**
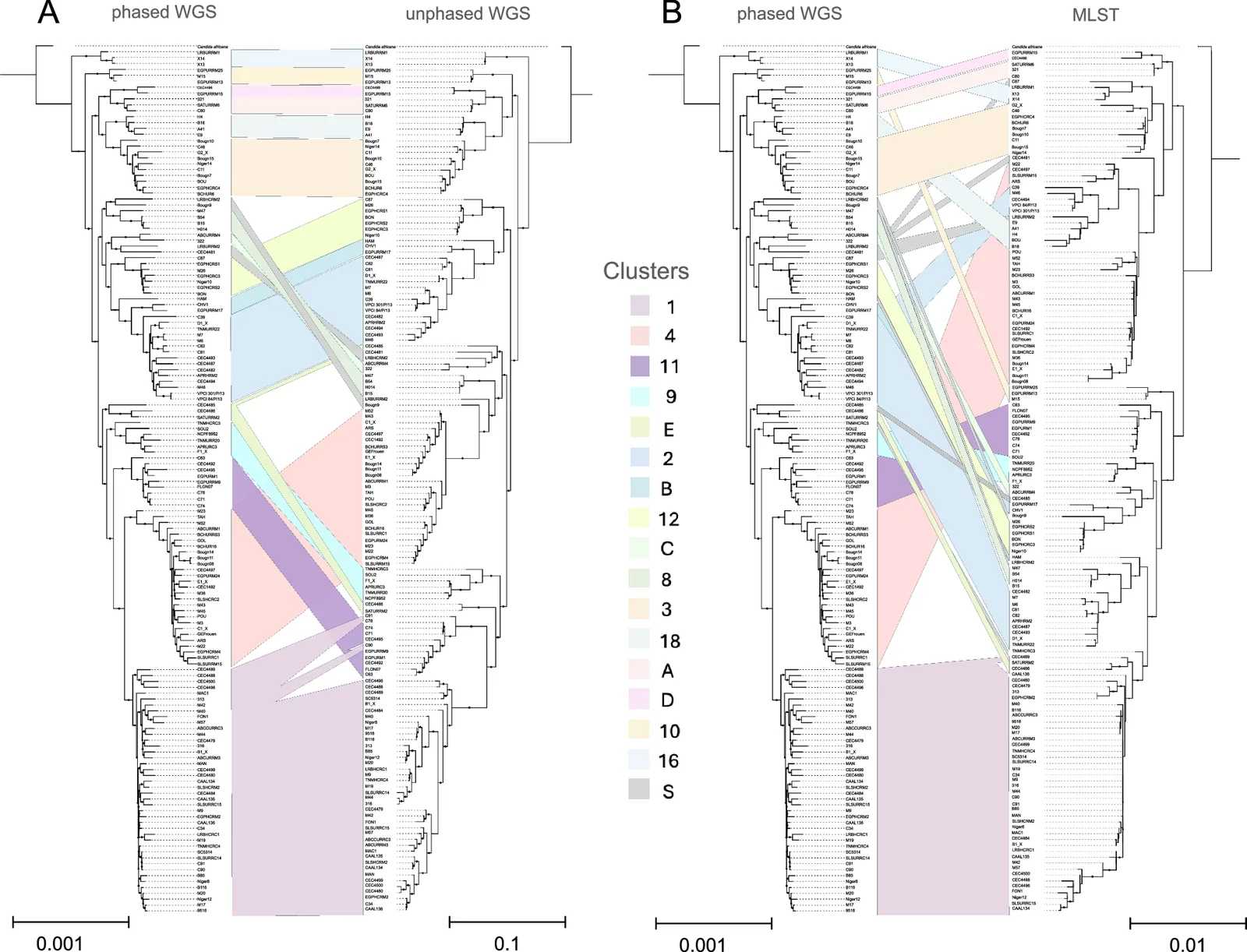
Topological differences in within-species phylogenies created with different sequence analysis methods. On the right side of both panels is phased short-read WGS data. This is compared to **A** a phylogeny constructed from unphased short-read WGS data, and **B** a phylogeny constructed from MLST sequences. The color bands in both cases indicate a mismatch in the order of isolates and were drawn to assist with a visual interpretation of the differences.

A primary goal of this project was to create an updated intraspecific phylogeny for *C. albicans*. The existing WGS tree included short-read, unphased data from 182 isolates. To assess the impact of incorporating haplotype phasing information, we reconstructed this tree (“unphased WGS”) and compared it to trees with the same isolates with haplotype phasing (“phased WGS”) and a multilocus sequence typing tree (“MLST”). The phased tree was constructed with 2,252,881 SNPs, approximately twice the number used in the unphased tree (1,113,404 SNPs), while the MLST tree relied on 197 SNPs. The majority of nodes in all generated trees have bootstrap support above 80% (Fig. 2). The normalized Robinson-Foulds (nRF) distance (a measure of topological dissimilarity between trees) was 0.655 betweenthe phased and unphased WGS trees, while the nRF distance between the phased WGS tree and the MLST tree was even higher (0.862). Taken together, this demonstrates that including haplotype information (and, accordingly, approximately double the number of included variants) influences the tree topology, as seen by visually comparing the trees (Fig. 2). Although the MLST tree partially recapitulates the WGS tree and the overall patterns of genetic divergence, it lacks the phylogenetic resolution provided by genome-wide data, suggesting that MLST analysis in *C. albicans* should be used with caution when precise phylogenetic relationships are desired.

### Updated *C. albicans* phylogeny

As haplotype phasing affects both tree topology and branch lengths, we therefore constructed the updated global whole-genome phylogeny using phased genomes from the full (1178) isolate set. Once the initial phylogeny was generated, we visually assessed the 361 intrapopulation isolate set that included multiple isolates from the same person. For each of the 95 people with multiple isolates, we determined the minimum number of isolates to retain to remove monophyletic isolate clusters from the same person; we retained 128 intra-individual isolates (Fig. S1). The vast majority of removed isolates came from North America (*n* = 217). The 35 isolates identified as cluster 13 (*C. africana*) in the existing WGS tree, along with two additional isolates that grouped with them, were included as the outgroup. The final tree is thus comprised of 908 *C. albicans* “phylogenetically informative” isolates (and 37 *C. africana*), which we refer to as the “phylogenetic informative set” of isolates (Table S2).

The overall topology of the final phylogeny largely, though not entirely, recapitulated the existing WGS tree clusters (Fig. S2). Five isolates from the existing WGS tree clustered differently: the two cluster B isolates clustered with cluster 2 isolates, the two cluster D isolates clustered with cluster C, and one cluster 3 isolate clustered with cluster 1. In the much larger strain set, all ten singletons had closely related isolates. This discrepancy could be due to differences in the isolate set or to the use of approximately twice as many variants in constructing the phased tree compared to the existing unphased WGS tree.

**Fig. 3:**
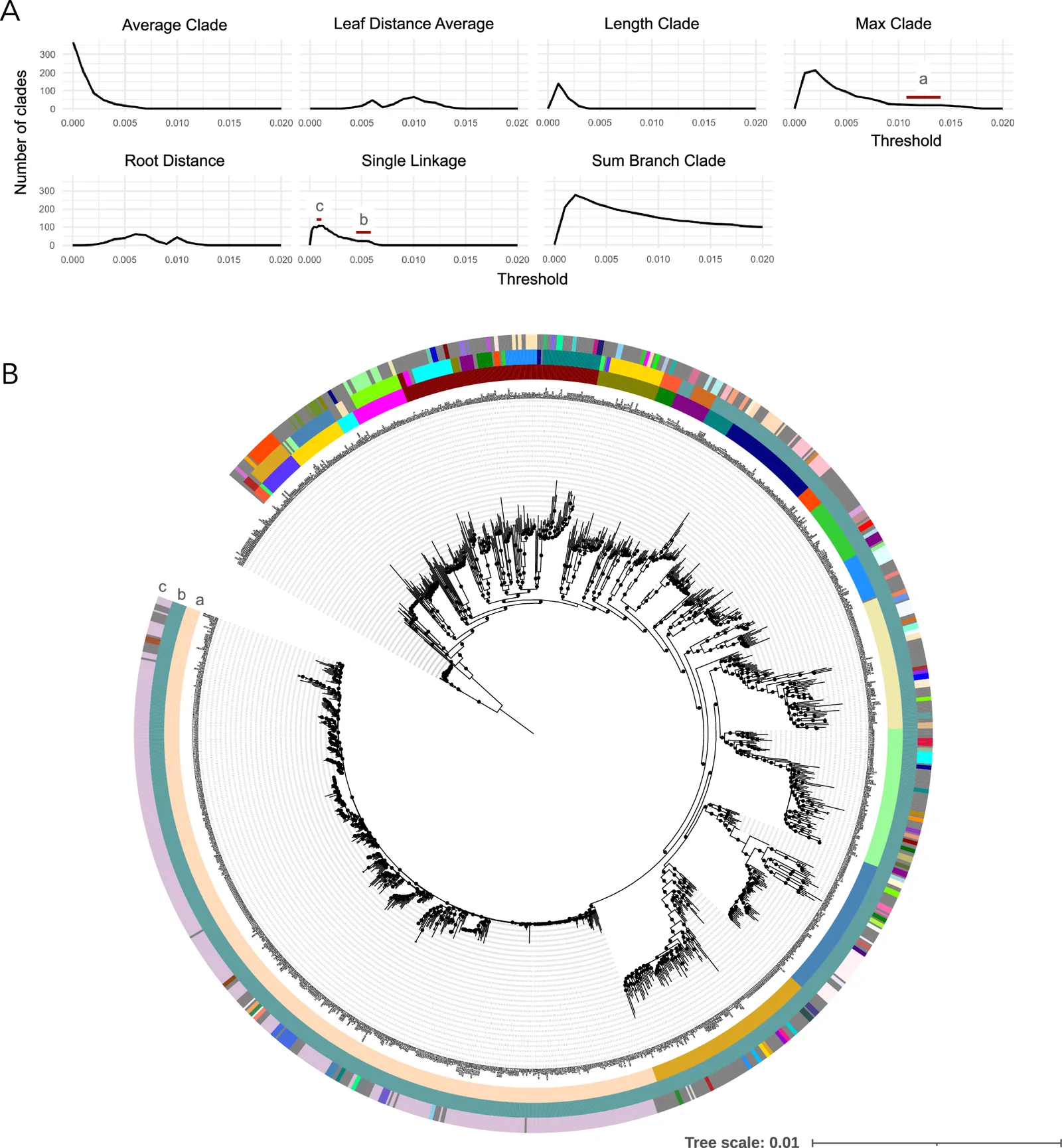
Clusters delineation using TreeCluster across multiple methods. **A** The number of predicted genetic clusters across a range of threshold values (0 to 1, in 0.001 increments) using seven TreeCluster methods. Regions with stable cluster predictions, defined as consistent cluster sizes across multiple consecutive thresholds, are highlighted with a dark brown bar and labeled a–c. These include one max clade cluster (20 clusters, thresholds t = 0.012–0.014, a) and two single linkage clusters (24 clusters, b, and 109 clusters, c). **B** Visualization of the stable clusters identified in (**A**) mapped onto the phylogenetic tree. Single linkage at a value of 0.009, which identified 109 clusters, was examined to visualize one of the high-cluster-number models and was not otherwise considered.

To delineate clusters for the updated tree, we sought to adopt a statistically supported approach that could be easily replicated on an extended or different strain set. We implemented seven TreeCluster strategies that determine phylogenetic tree topologies by clustering tip sequences with different optimization functions and distance thresholds. Given that isolate designations have been relatively consistent over time across different sequencing technologies, we expected to find regions of threshold space that would yield approximately 17 clusters (the number from the existing WGS tree) with substantial overlap in cluster assignments. Five strategies showed no threshold regions of cluster-number stability, with the majority of values at either a single cluster or very high (>50) numbers of clusters (Fig. 3A). Two strategies, however, yielded broad regions of parameter space where the same number of clusters was identified (Fig. 3A). The default, “max clade” strategy, identified 20 clusters from threshold parameter space values between 0.012–0.014, while the “single linkage” strategy (which has previously been used in HIV research) identified 24 clusters over values 0.047–0.055, excluding 0.051. However, the 24 clusters suggested by the single linkage strategy combined approximately 3/4 of the isolates into a single cluster, with all additional clusters identified among the isolates closest to the root of the tree (Fig. 3B).

**Fig. 4:**
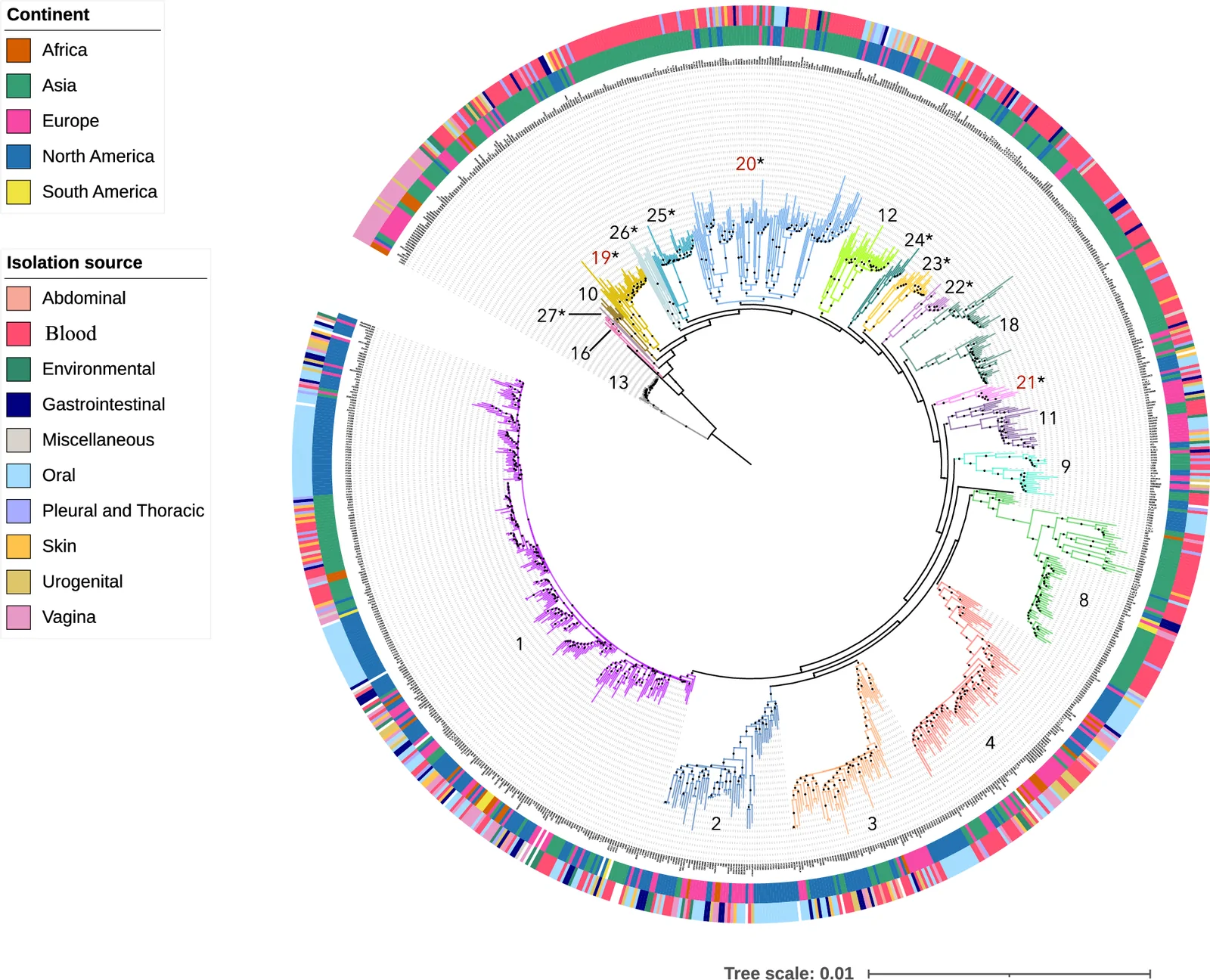
Maximum likelihood phylogeny of 945 isolates. Branch colors correspond to cluster designations as defined in the study. Cluster numbers are shown near the branch tips, and asterisks indicate new cluster labels. The red clusters are labels given to clusters from the existing WGS tree that had been assigned letters. Bootstrap support values ≥80% are indicated by dots on the internal branches. Two concentric rings surround the phylogeny: the inner ring represents the continent of origin for each isolate, while the outer ring denotes the anatomical source of isolation. This visualization highlights both the phylogenetic structure and the geographic and ecological diversity across clusters.

When we mapped the max clade cluster designations onto the final phylogeny, it was visually clear that most clusters matched the existing WGS tree well (Fig. 4). Fourteen clusters from the existing WGS phylogeny were retained. Both clusters with previous evidence of gene flow from the admixture analysis in the existing WGS tree were merged into one of their ancestral parent clusters (cluster B was nested into cluster 2, and cluster A was nested into cluster D). We first assigned new numeric designations to the three existing clusters that had previously been assigned letters (cluster A/D was renamed to cluster 19, cluster C to cluster 20, and cluster E to cluster 21). In addition, six novel clusters were identified that were not present in the existing WGS tree; these were numbered 22–27 based on their phylogenetic location in a counter-clockwise manner (Fig. 4).

We extracted the MLST sequence information from the phylogenetically informative isolates and generated a MLST phylogenetic tree. Although many isolates from the same WGS-defined clusters grouped together in the MLST tree, there were also many cases of discordance, where isolates from different WGS clusters grouped together, or isolates from the same WGS cluster were apart (Fig. S3). Six clusters defined in the MLST tree were absent in the existing WGS tree (clusters 5, 6, 7, 14, 15, and 17). When we overlay our WGS cluster designations with all of the available MLST sequence information (i.e., from our isolates as well as those from pubMLST)^40^, isolates from the absent MLST clades did not group with any of the new WGS clusters either (Fig. S4). As we lack WGS sequence information for these historical isolates, as in the existing WGS tree, we did not reassign those cluster numbers.

**Fig. 5:**
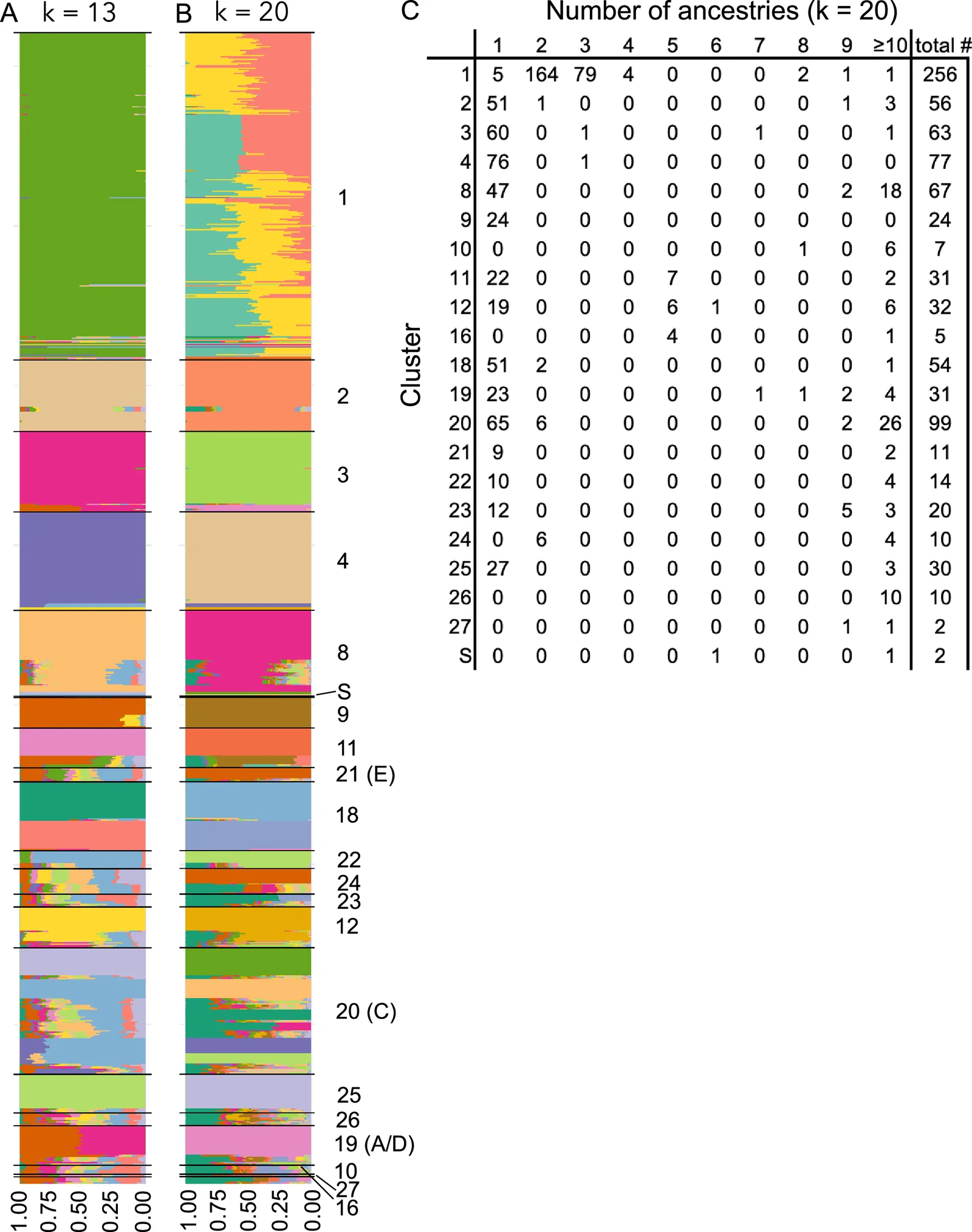
Population structure of the phylogenetically informative isolates inferred by NGSadmix analysis. NGSadmix ancestry profiles of isolates at **A** k = 13 and **B** k = 20. Each horizontal bar represents an individual isolate, ordered according to its position in the phylogenetic tree, and colored segments indicate the proportion of ancestry assigned to each inferred cluster. Horizontal black lines denote boundaries between proposed phylogenetic clades. **C** The table summarizes, for each clade, the number of distinct ancestry components detected among isolates at k = 20.

A previous admixture analysis of isolates included in the existing WGS tree identified two clusters containing isolates with multiple ancestries at k = 13^22^. In addition, a visual inspection of the existing admixture plot shows that the 10 isolates previously classified as singletons were also highly admixed. However, admixture signals observed in singleton or sparsely represented groups should be interpreted with caution, as populations with fewer than five individuals are known to yield unreliable ancestry estimate^41^. When we re-ran admixture analysis on the updated phylogeny from k = 6 to k = 20, a much higher proportion of isolates appears to be admixed than previously identified (Figs. 5 and S5). At k = 20 (i.e., the same number of ancestries as statistically identified clusters), 60% (*n* = 501) of the isolates were identified as single ancestry, while 20% (*n* = 140) of the isolates were highly admixed (from 4–18 ancestries). Interestingly, at either k = 13 (Fig. 5A), or k = 20 (Fig. 5B), the majority of clusters that were previously identified to contain almost entirely single-ancestry isolates either continued to show this (clusters 2, 3, 4, 9, 18, 21), or contained subclusters, a large one with single-ancestry isolates and a smaller one with mixed-ancestry isolates (clusters 8, 11, 12, 22, 23; Fig. 5C). The exceptions were the isolates in cluster 1, which at k = 20 (but not k = 13), had either two or three ancestries, isolates in cluster 10 (which were all highly admixed at all k values), and isolates in cluster 20 (formerly cluster C), which contained several blocks of highly admixed isolates. Whether cluster 1 contains predominantly single- or mixed-ancestry isolates depends heavily on k: most k values below 13 classify the isolates as single ancestry, whereas higher k values classify them as mixed ancestry (Fig. S5). Cluster 19, which contains the isolates previously classified as mixed ancestry in cluster A, is similar to cluster 1, with isolate classification (single or of two ancestries) depending on the chosen k value (Fig. S5). The six new clusters were split between those with highly admixed ancestry isolate subclusters (clusters 22, 23, 25) and those containing entirely highly admixed isolates (clusters 24, 26, and 27).

### Geography and site of isolation are nested together

The distribution of isolates in the full phylogeny was largely consistent with the existing WGS tree. Cluster 1 contained approximately a quarter of all isolates, while three clusters (10, 16, 27) had fewer than ten isolates. Cluster designation, isolation source (Figs. S6 and S7), and geographic origin (Fig. S8) are not independent of each other. This is seen visually on the map (Fig. 1) and the overlapping color blocks in the outer rings of the phylogeny (e.g., North American oral isolates exhibit several clean blocks in multiple clusters; Fig. 4). There was a significant statistical association between all three pairwise factor comparisons, though the association between isolation source and continent had the largest effect size (pairwise Chi-square tests with 100,000 Monte Carlo simulations; source × continent: χ^2^ = 1064, *p* < 0.0001, Cramér’s V = 0.49; cluster × source: χ^2^ = 433, *p* < 0.0001, V = 0.23; cluster × continent: χ^2^ = 473, *p* < 0.0001, V = 0.33). Isolates from the blood were overrepresented in Asia relative to North America (and, to a lesser extent, Europe), whereas oral isolates were similarly overrepresented in North America relative to Asia (Fig. S9). Interestingly, Africa and South America are both overrepresented in vaginal isolates, while Europe is overrepresented in environmental and urogenital isolates (Fig. S9). These results suggest that while there is some significant phylogenetic structure based on geography and isolation source, these factors are currently largely confounded and heavily influenced by research focus.

### Manitoba as a case study to remove geography

To examine the phylogenetic relationships among isolates from different sources with the potential confounding factor of geography removed, we analyzed a set of 83 isolates collected from a hospital microbiology lab in Manitoba (this set entails all *C. albicans* isolates collected in 2012 and 2018). The isolates fell into nine clusters, eight of which were present in both years; there was no significant difference in cluster composition between the two years (χ² = 6.64, *p* = 0.6534; Fig. S10). The isolates were obtained from nine isolation sources; notably, no significant association was observed between cluster and isolation source (χ² = 66.90, *p* = 0.3814), and isolates from different sources (and different years) often grouped right beside each other. This, as previously shown, highlights that there is no clear pattern between cluster and isolation source, and that global migration appears relatively common among *C. albicans* genotypes. However, this picture remains incomplete, as a signature of geographic enrichment is present in many clusters. Indeed, the novel clusters we identify in our strain set, relative to the existing WGS tree, are largely composed of Asian isolates, a region that was poorly represented in the existing WGS tree. Combining the global and local (Manitoba) analyses suggests that geography, rather than the isolation source, has a greater impact on shaping phylogenetic relationships in *C. albicans*.

### Karyotypic variation

**Fig. 6:**
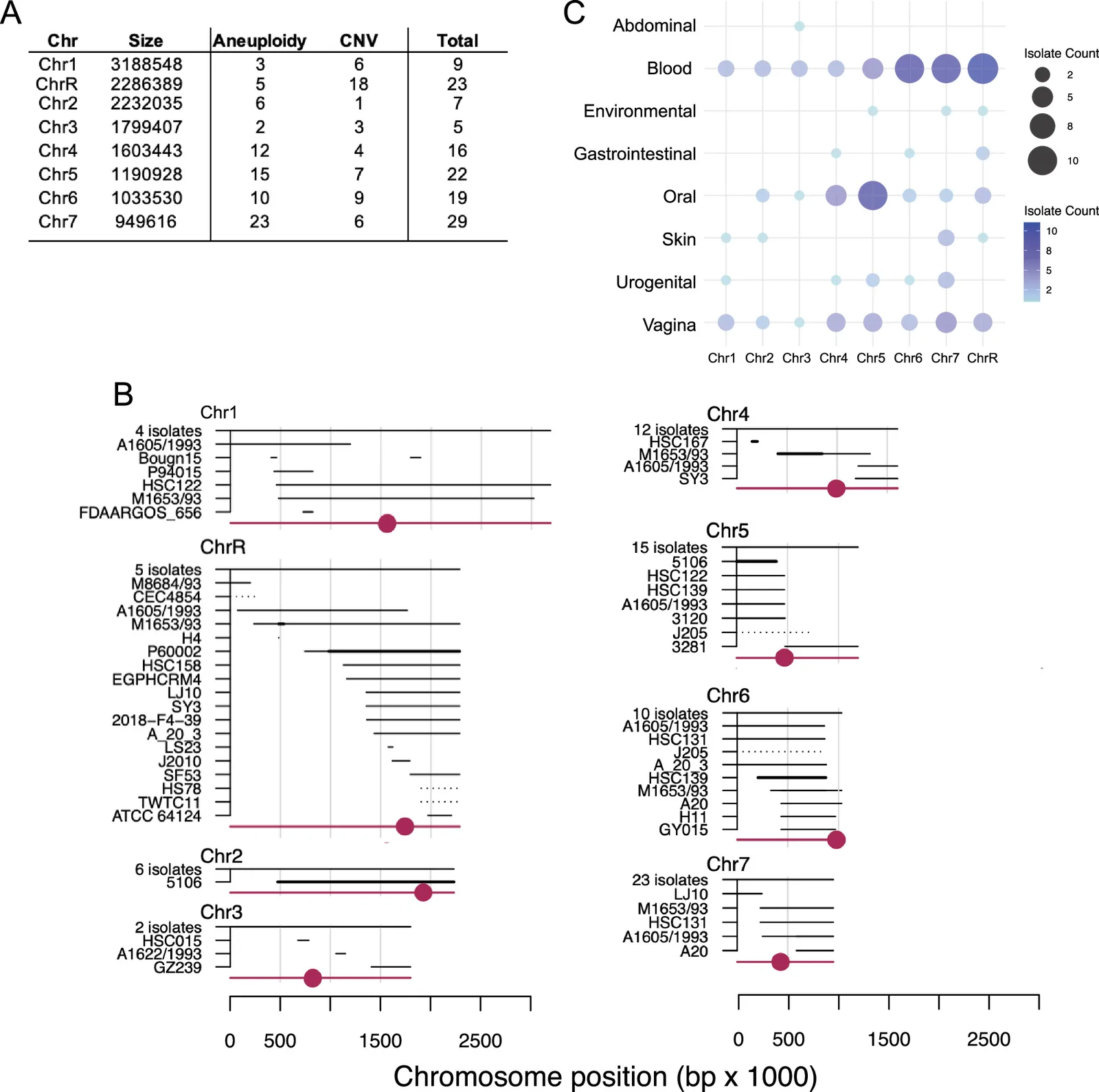
Karyotypic variation among isolates. **A** Distribution of aneuploidies and copy number variations (CNVs) across chromosomes. Karyotypic variation is more frequent in smaller chromosomes, suggesting that structural alterations may be better tolerated in shorter chromosomal regions. **B** Observed aneuploidies and CNVs in individual isolates, organized by chromosome. The length of each line represents the genomic span of the event, while the thickness indicates the magnitude of the duplication or deletion. **C** Bubble plot showing the distribution of aneuploid isolate counts across chromosomes and isolation sources. Each bubble’s size and color represent the number of isolates detected for a specific chromosome-source combination. Larger and darker bubbles indicate higher counts.

The full isolate set was examined for aneuploidies and copy number variations (CNVs; Fig. S11). 86 isolates exhibited karyotypic variation: 57 isolates had at least one aneuploidy, 30 isolates had at least one CNV region larger than 50 kb, and six isolates had both (Fig. 6A). Four of these isolates were identified as triploid (3 N), four as tetraploid (4 N), and the base ploidy could not be determined for four isolates. All of the triploid isolates had multiple aneuploid chromosomes, while the tetraploids had only a single one. The use of WGS coverage to determine ploidy precludes the identification of euploid polyploids, so this represents a lower limit for ploidy variants in the strain set. There was a significant negative correlation between chromosome size and the number of aneuploidies (Pearson’s correlation: t_6_ = −2.80, *p* = 0.031, cor = −0.75), potentially indicating less constraint against aneuploidy on smaller chromosomes that carry fewer genes. The exceptions were chromosomes 3 and 6, which both had fewer aneuploidies than their size would have predicted, suggesting there might be stronger selection against extra copies of these chromosomes. There was a pattern of association between specific aneuploidies and the site of isolation: blood isolates were most likely to have a CNV or aneuploidy of chromosomes 6, 7 and R; oral isolates were most likely to have karyotypic variants on chromosomes 4 or 5; vaginal isolates had relatively equal distribution. There was no correlation between chromosome size and the number of CNVs (t_6_ = 0.11, *p* = 0.92), nor between the number of aneuploidies and the number of CNVs (t_6_ = −0.11, *p* = 0.91). The majority of CNVs across all chromosomes were terminal, in many cases in both directions (i.e., CNVs that extended to the telomeres), though for most chromosomes a small number of interstitial CNVs were also observed (Fig. 6B). Some regions seemed more likely to be involved in a CNV (e.g., regions of overlap among many CNVs on chromosome 6 and chromosome R), and CNVs in general were far more common on chromosome R than other chromosomes (Fig. 6B).

Karyotypically-variant isolates were observed throughout the phylogeny and were typically not clustered together (Fig. S11). At least one isolate from each isolation source was observed (Fig. 6C), with the number proportional to the total number of isolates from each source. The three most common sources exhibited different patterns, which hints at a potential beneficial association between selection in these different niches (blood versus oral) selecting for different chromosomal variants. However, caution in interpretation is needed given the sampling biases. The bloodstream isolates are from multiple locations and published sources, while the oral isolates all come from a single study of North American isolates focused on drug resistance^42^.

### *MTL* locus analyses

The distribution and variation of mating-type loci (*MTL*) were examined in the phylogenetically informative set of isolates (Table S1 and Fig. S11). Read alignments to the A22 reference genome (A22-s08-m01-r09 from the Candida Genome Database) were used to assess coverage across the *MT*L**a** and *MTL***α** regions, with regions showing coverage below 0.5 interpreted as inactive or absent. As expected, the predominant genotype was the heterozygous diploid a/α, observed in 850 of 908 isolates (93.6%). Other genotypes were rare but present, including a/a (1.9%), α/α (3.5%), and triploid *MTL* configurations in chromosome 5 aneuploids (a/a/a: 0.1%; a/a/α: 0.4%; a/α/α: 0.3%; α/α/α: 0.1%). Homozygous *MTL* isolates were from every continent and all major isolation sources. A statistically significant but modest association was observed between *MTL* genotype and continent (Fisher’s exact test with Monte Carlo simulation, *p* = 0.016) and MTL genotype and cluster (*p* = 0.012), while there was no association between *MTL* genotype and isolation source (*p* = 0.075). That isolation source was not significant suggests that neutral processes likely drive variation in the distribution of *MTL* genotypes.

### Heterozygosity analyses

**Fig. 7:**
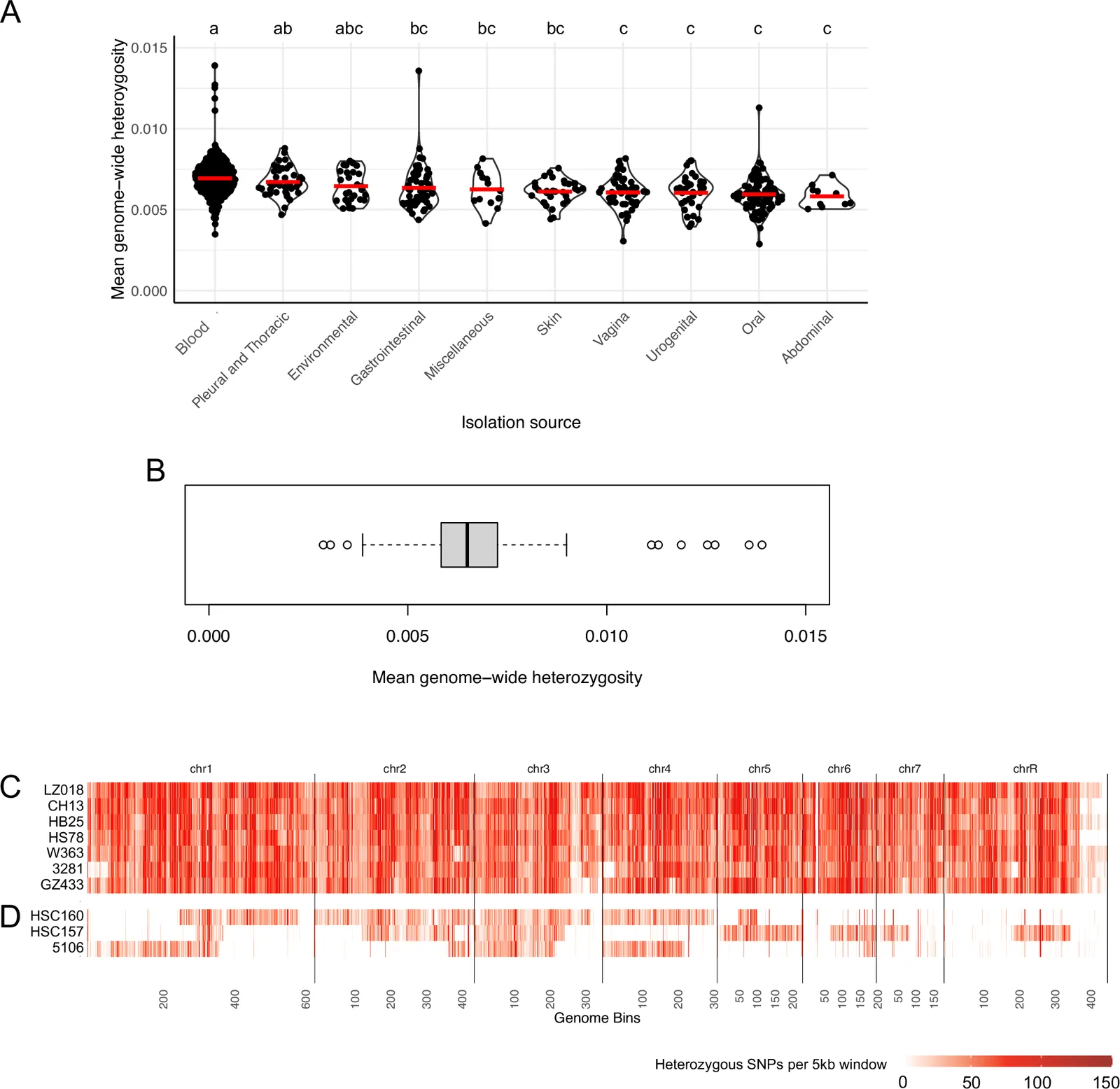
Genome-wide heterozygosity among isolates. **A** Average genome-wide heterozygosity varies across different isolation sources. Letters above each violin plot indicate statistically significant differences based on a post hoc Tukey’s test following a two-factor ANOVA; groups that do not share a letter differ significantly. **B** Boxplot showing the distribution of average genome-wide heterozygosity across all isolates. **C**, **D** Density plots of the number of heterozygous SNPs in 5 kb windows across the genome. Each row represents an isolate, and vertical black lines indicate chromosome boundaries (chromosomes 1–7 and R). The scale bar reflects the density of heterozygous SNPs per 5 kb window, from none (white) to high density (dark red). **C** Outlier isolates with high average genome-wide heterozygosity. **D** Outlier isolates with low heterozygosity, largely due to large LOH regions.

Heterozygosity was assessed across the 744 isolates with genomic information at ≥85% of sites. The mean genome-wide heterozygosity was 0.0065 ± 0.001 (SD), corresponding to approximately 6.5 SNPs/kb, and ranged from 0.0029 to 0.014 (2.9–14 SNPs/kb). To examine the potential effects of different factors on genome-wide heterozygosity, we conducted a three-factor ANOVA with isolation source, cluster, and geography as factors. There was a significant interaction between isolation source and cluster in genome-wide heterozygosity across all isolation sources, while geography was not significant as either a main effect or in an interaction term. We thus dropped geography and re-ran a two-factor ANOVA, finding that both factors were significant as both main effects and an interaction (Two-factor ANOVA test; isolation source: F_9, 615_ = 17.4, *p* < 0.0001, cluster: F_20, 615_ = 14.5, *p* < 0.0001, isolation source × cluster: F_85, 615_ = 1.7, *p* < 0.0001, Fig. 7A). Post hoc comparisons identified that blood isolates had significantly higher heterozygosity than isolates from many other sites, though the distributions across different sites were highly overlapping (see Fig. 7A for statistical results). Analysis of heterozygosity across the eight chromosomes revealed variability in mean values, with chromosome 6 exhibiting the highest average (0.00814 ± 0.00324; ~8.14 SNPs/kb) and chromosome R the lowest (0.00572 ± 0.00152; ~5.72 SNPs/kb) (Table S2).

**Fig. 8:**
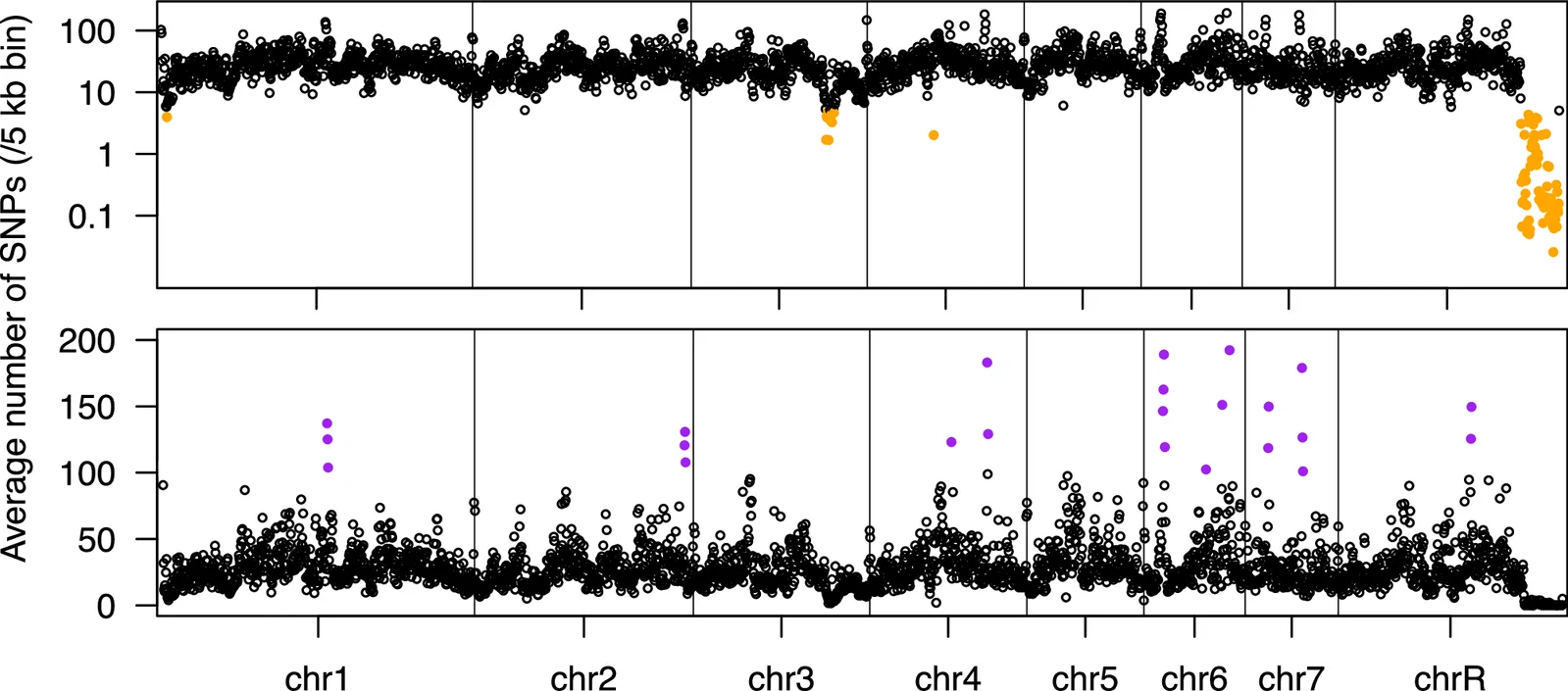
Genome-wide distribution of the average number of heterozygous SNPs across 745 *C. albicans* isolates. Low-heterozygosity regions (defined as <5 SNPs per window) are shown as orange circles (upper panel), while high-heterozygosity regions (defined as >100 SNPs per window) are indicated by purple circles (lower panel).

Although most isolates had a similar level of genome-wide heterozygosity, several isolates were significantly lower or higher than average (Fig. 7B). There were seven highly heterozygous isolates. Six of the seven originated from China, including five bloodstream isolates and one gastrointestinal isolate, while the remaining isolate was an oral isolate from the United States. Although these seven isolates are admixed at K = 13 and K = 20, highly admixed ancestry profiles do not uniformly correspond to the highest levels of heterozygosity, indicating that admixture alone is insufficient to explain the observed heterozygosity. By contrast, the three low-heterozygosity isolates are all single-ancestry isolates from three different sources (oral, vaginal, blood) and three different clusters (2, 3, 20) from North America. To determine whether specific regions of the genome were causative, for each isolate we divided the genome into 5 kb bins and counted the number of heterozygous positions in each. There were many small LOH events across the isolates. The LOH regions encompassed regions common to all isolates within clusters, later events that are common to only some members of a cluster, and isolate-specific events (Fig. S12). Notably, the high-heterozygosity isolates contained heterozygous positions throughout the genome (Fig. 7C). However, the terminal end of chromosome R was consistently depleted for heterozygosity across most isolates, including the highly heterozygous isolates^22^. This suggests that there was either an ancestral LOH event prior to the most recent common ancestor of the species, or that there have been several recurrent LOH events extending from the rDNA toward the telomere, a pattern that was previously observed in *S. cerevisiae*^43^ and thought to be driven by elevated recombination at the rDNA promoted by a conflict between transcription and replication (Figs. 8 and S12). Surprisingly, given the small sample size, the low-heterozygosity isolates exhibit distinct patterns across the genome, with nearly all chromosome arms (except chromosome 3) represented in reduced-heterozygosity regions.

To look for common regions of both high and low heterozygosity, we examined the average heterozygosity within each 5-kb bin across all isolates and examined the resulting histogram of the regions. Based on this, we defined regions of low heterozygosity as <1 heterozygous base/kb. This identified 119 bins; the majority (*n* = 78) are at the terminal right end of chromosome R. Three additional regions are located on chromosomes 1 (one bin), 2 (one bin), 3 (twelve bins), 4 (one bin) and 5 (two bins, Fig. 8). Many named genes with known or predicted functions that could be the target of purifying selection are present (Table S3). There were overall fewer bins with elevated heterozygosity, defined as those with >100 heterozygous bases on average (i.e., heterozygosity > 0.1/kb); they were found in 10 distinct genomic regions distributed across five chromosomes (Fig. 8). After excluding bins that include annotated repeat sequences, we identified 12 unique protein-coding genes within these variable regions. The genes included *HAL21*, *CAN1*, *RIM9*, *KAR5*, and *MSY1*, along with several encoding proteins with unknown functions (Table S3). Further work is required to validate whether there is a fitness benefit to increased heterozygosity or disruptive selection (e.g., favoring variation among isolates in different ecological contexts) in these genes.

### Ago1 PAZ domain analyses

**Fig. 9:**
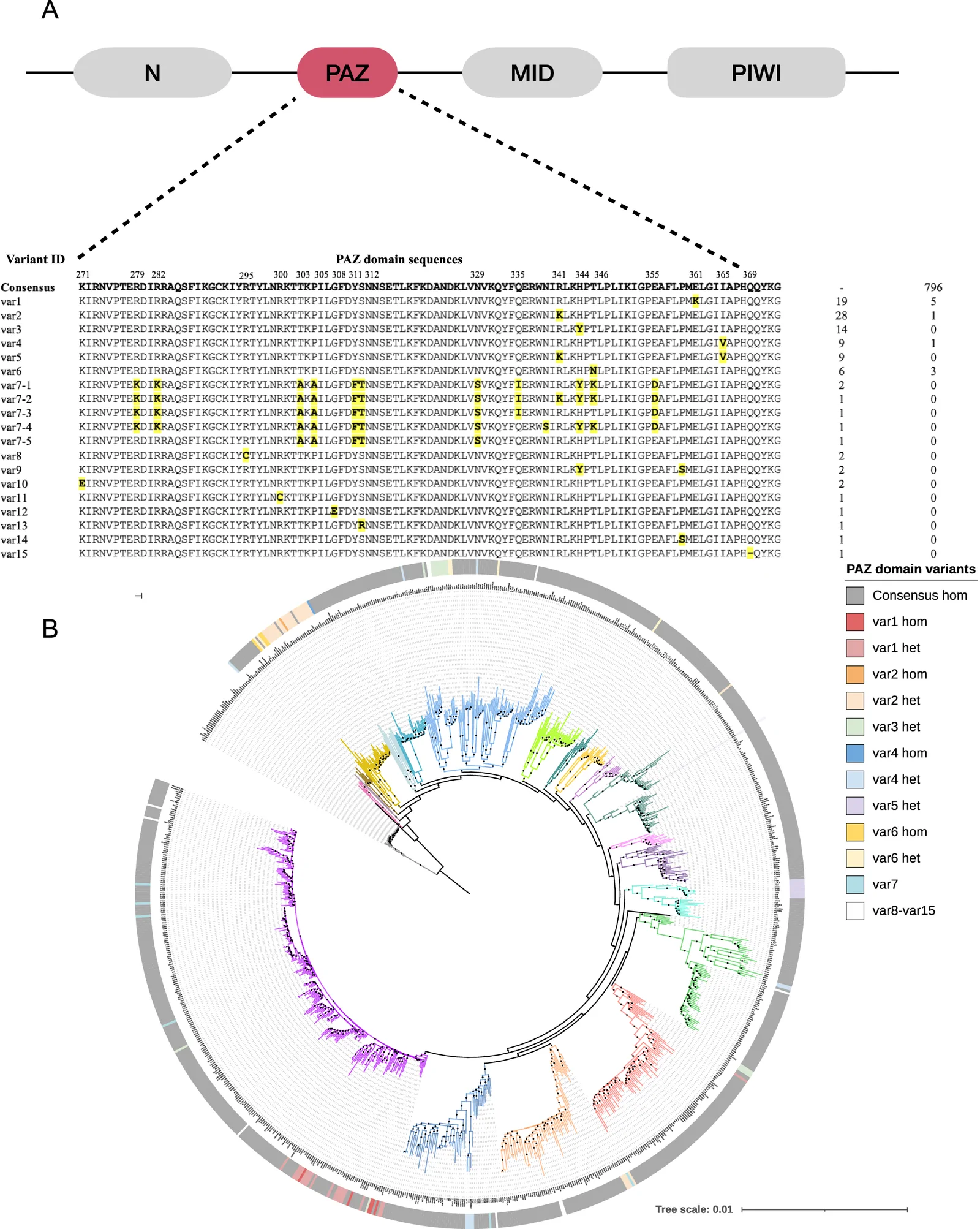
*Candida albicans* Ago1 PAZ domain sequence variants. **A** Schematic representation of *C. albicans* AGO1 protein and alignment of unique PAZ domain sequences (aa 271–373) identified from 908 *C. albicans* isolates with the consensus (known active) sequence in bold. Positions that differ from the consensus domain are highlighted in yellow, with the positions indicated in the column. **B** The distribution of the variant in the phylogeny. The branches of the phylogeny are colored by cluster.

We aimed to determine how widespread the RNAi-deficient genotype observed in the SC5314 reference strain is among other *C. albicans* isolates. Among the three canonical domains conserved in Ago proteins (PAZ, MID, and PIWI), we focused on the PAZ domain, which was the one previously shown to be mutated in the SC5314 reference strain. We identified 43 unique SNPs in the PAZ domain among the phylogenetically informative set of 908 *C. albicans* isolates (excluding cluster 13 isolates): 17 were synonymous, and 26 were non-synonymous (Fig. 9).

The RNAi-active consensus PAZ sequence was homozygous in 796 isolates (88%), distributed across the phylogenetic tree (Fig. 9A, B). Nineteen distinct PAZ domain variants (sequences) containing between 1 and 2 amino acid changes were identified. We identified three additional homozygous variants in the PAZ domain, in addition to the previously described var1 (K361; *n* = 5): var2 (K341; *n* = 1), var4 (N346; *n* = 1), and var6 (V365; *n* = 3). Only these four variants (var1, var2, var4, and var6) were observed in a homozygous state, indicating that the wild-type PAZ domain sequence was retained in the remaining 898 isolates. Var1, the variant found homozygous in SC5314, was restricted to five additional cluster 1 isolates, while a heterozygous version of this allele was found in 19 additional cluster 1 isolates and one cluster 4 isolate (Fig. 9B). The var2 homozygous variant was found in a single cluster 19 isolate, with the heterozygous form most commonly found in the same cluster. Other variants, although heterozygous, showed cluster-specific enrichment: var3 was predominant in a subcluster of cluster 20, whereas var5 was mainly associated with cluster 9.

### Intra-individual analysis

Nine studies included more than one isolate collected from the same individual or from a healthy mother-infant dyad (Table S4). Nearly all individuals were from the United States (75) and Canada (12), four were from Spain, and one each from Morocco, France, Brazil, and Tunisia. The largest study included oral isolates from 59 mother-infant dyads in the United States. The other studies took multiple isolates at a single time point at the same body site (12 individuals, blood, lung and oral isolates), a single time point at multiple sites (7 individuals with either oral and fecal or rectal and vaginal site pairs) or multiple time points from the same or different body sites (8 individuals with oral samples, one individual with urine samples, and one individual with blood and pleural samples). The number of isolates per individual ranged from 2 to 23 (mean 3.8, median 2). Most were colonized by genetically monophyletic *C. albicans* populations, though 14 individuals carried isolates from two clusters: ten from healthy mother-infant dyads, two from sequential oral isolates taken from HIV patients, one from sequential isolates taken from urine, and two from oral and fecal samples taken from healthy individuals. One individual contained isolates from three clusters (oral and fecal isolates from a healthy individual). More studies on intra-individual isolates (including isolates from the same site taken at the same time) are critically required to fully characterize intrapopulation diversity under different contexts.

### Environmental isolates

The 31 environmental isolates were collected from several sources, including food spoilage, tree bark, starling (bird) feces, soil, duff, and fruit. Most environmental isolates clustered closely with clinical isolates throughout the phylogeny (Fig. 4). However, one fruit isolate from Sand Island Marine Park in the United States (a ~0.13 km^2^ offshore island in the Columbia River close to the border of Oregon and Washington State) was a singleton that did not cluster with any other isolates and was an outgroup to the rest of the *C. albicans* phylogeny. The two European isolates from starlings were positioned adjacent to the outgroup in cluster 16, which has only three other isolates (a urogenital isolate from Europe, an oral isolate from the UK, and a blood isolate from China). This suggests they fall into one of the earliest-diverging lineages among the sampled isolates.

The average heterozygosity of environmental isolates did not differ significantly from other sources (Fig. 6A), nor were they more likely to be aneuploid, MTL homozygous, or Ago1 variant. As 19 of the environmental isolates are from food spoilage, the true environmental representation in the phylogeny is very small. Given that three of the ‘true’ environmental isolates are found near the outgroup, this suggests that additional effort in environmental sampling and sampling non-human hosts is needed to better capture the true breadth and diversity of environmental isolates specifically and *C. albicans* diversity generally.

## Discussion

The distribution of *C. albicans* isolates whose genomes have been sequenced using short-read technologies reveals significant sampling biases in both geography and source of isolation. There is a notable absence of large-scale, region-specific studies that capture isolate diversity from Africa, the Middle East, and Australia, and the few isolates that do exist are only from a small number of countries. Similar to the disparity among WGS isolates, among the ~6000 isolates submitted to the pubMLST database (as of July 30, 2025), only 197 are from Africa and 88 from Oceania^40,44^. The lack of genomic studies from these locations, which exhibit some of the highest global diversity in other taxa, highlights a critical gap in our ability to capture local and global *C. albicans* intraspecific diversity and negatively impacts our ability to assess regional genetic diversity and population structure.

The types of studies that have generated WGS data are also biased, often focused on specific disease presentations. For example, a large proportion of isolates come from studies on candidemia (particularly from Asia^45,46^), oral candidiasis (particularly from North America^14,42^), and commensal colonization^14,47,48^. Vaginal isolates are the most evenly sampled across continents, consistent with this being the most common disease presentation. Environmental isolates are rare, and only a handful of isolates have been collected from genuinely natural environments, such as tree bark^10^, soil^11^, duff^11^, and fruits^11^, rather than clearly human-associated environments, such as food spoilage^20^. Despite their scarcity, the environmental isolates generally show high genomic similarity to isolates from human-associated isolates, though several do seem to contain a unique or nearly unique phylogenetic signal. The three oak tree isolates exhibited either similar or higher host-damage potential and intrinsic resistance to amphotericin B and fluconazole compared to clinical isolates, indicating that environmental *C. albicans* can harbor substantial pathogenic and drug-resistant traits^49^.

*C. albicans* has been shown to be transmitted through vertical (mother-to-child)^12–14^ and intrafamilial transmission^50^. Isolates from the same geographic location, obtained from different isolation sources, often cluster together, suggesting the potential for extrafamilial routes of transmission. It would be very interesting to examine isolates over a longer period within the same human cohort to determine whether there is strain turnover in later life stages. There is a pressing need for expanded sampling across multiple scales to better understand the factors that drive relatedness in the *C. albicans* phylogeny.

We used phased whole-genome data, together with a statistical tool, to construct a phylogeny and identify 20 clusters. The phylogenetic structure is largely consistent with the existing WGS tree^20^, while identifying six additional clusters. The isolates in the new clusters are predominantly from Asia. A recent study from China that added 369 isolates to the existing WGS tree proposed 38 clusters, including 21 novel clusters^46^. In our analyses (which also included these isolates), the novel clusters described by Gong et al. were either merged into existing clusters or were classified as one of the six novel clusters we described. Schikora-Tamarit and Gabaldón (2024) also identified four additional clades in their analysis. The identification of additional clades by these studies supports the view that there are isolates from additional clusters that were not represented in the existing WGS tree^27^.

From the historical MLST cluster nomenclature, six cluster numbers are unaccounted for in our phylogeny (clusters 5, 6, 7, 14, 15, 17). None of the novel WGS clusters corresponds to these missing MLST clusters. Unfortunately, no WGS data was available for isolates from those clusters, which should be prioritized for short-read sequencing in the future. We proposed an expanded cluster designation for new WGS clusters with numbers starting at 19, including a revision of the alphabetic cluster nomenclature introduced by Ropars et al.^20^ to a fully numeric system. This approach standardizes clade nomenclature, minimizes confusion across studies, and conservatively maintains continuity with prior work. Under this framework, we recognize the potential for 27 identified clusters (20 from our WGS tree and up to seven from the MLST legacy isolates). Clusters 24, 26, and 27 consisted entirely of admixed isolates. Further investigation using approaches designed to characterize admixture and reticulate evolutionary histories may help clarify their origins.

The lack of strong geographic or isolation source-specific patterns in chromosomal aneuploidy and copy number variations (CNVs) suggests that these genomic alterations likely arise from local or individual-level selective pressures, rather than being driven by broader population structure or regional factors. We found that 9.47% of isolates had a major karyotypic variation, which is very similar to the frequency reported by Ropars et al. for their strain set. The specific chromosomes involved differed between blood and oral isolates (chromosomes 6, 7, and R for blood; chromosomes 4 and 5 for oral). Trisomy of chromosome 5 is thought to be selected for during oropharyngeal candidiasis, as it facilitates a commensal-like phenotype^51^. However, studies have also shown that chromosome 7 trisomy can enhance colonization of both the gastrointestinal tract and the oral cavity in mouse models^52–54^. It may also be that some or many of the observed karyotypic variants are neutral or depend heavily on the genetic background. Karyotypic variants were observed throughout the phylogeny and were unclustered, supporting the idea that aneuploidy and CNVs are transient. It is also likely that their effects (if there are any) are strain-dependent. For example, although trisomy of chromosome 4 was shown to confer fluconazole resistance in a clinical *C. albicans* isolate^55^, aneuploidy was also observed in isolate T118 during serial passage in the presence of fluconazole, yet did not directly contribute to enhanced drug resistance^56^.

The distribution of mating-type locus (*MTL*) genotype was also relatively consistent with previous large-scale surveys. The homozygous mating-type loci were relatively rare (2.7%) and within the range of previously reported homozygosity rates of 2.2%^20,57^ and 3.2%^20,57^ (though Odds et al. observed a slightly higher prevalence of 8.5%^22^). In all studies, including this one, the α/α genotype was more common than the a/a genotype, suggesting that loss of the *MTL****a*** locus may occur more frequently or be more tolerated in the *C. albicans* population.

The average genome-wide heterozygosity among our isolates was 0.0065 (6.5 heterozygous SNPs per 1000 bases). This was nearly identical to the average of three isolates from oak trees (0.0066)^10^ and an analysis of 61 diverse isolates (0.0067)^58^, while higher than the average from Ropars et al. (0.0048)^20,57^. This is likely due to differences in methodology, rather than strain-set.

We found heterozygosity varied across anatomical sources, indicating that host environments may influence genetic diversity. Blood isolates, for example, displayed higher heterozygosity than gastrointestinal or oral isolates, potentially reflecting the immune-challenged and dynamic nature of the circulatory system. Lower heterozygosity in isolates from localized infections, such as abdominal or gastrointestinal sources, suggests more stable pathogen populations in these sites. LOH analyses revealed both ancestral and clade-specific events, including a conserved LOH region on chromosome R and clade-restricted LOH on chromosome 3 (clades 1, 4, 8). Differences in heterozygosity among *C. albicans* clades have previously been reported^20,46,59^, yet these differences are potentially driven by biased sampling, with the confounding variable of isolation source nested into clade.

The majority of isolates (>99%) maintained the RNAi-active consensus PAZ sequence in at least one copy, indicating that RNAi activity is likely preserved in the vast majority of *Candida albicans* isolates. However, we identified novel genetic alterations in the PAZ domain. A previous study examined 296 isolates and identified a single homozygous mutation (361 E/K) in the SC5314 reference strain and seven other isolates with RNAi deficiency^19^. They also identified isolates containing heterozygous variants at positions 345P and 365I that retained RNAi activity. In our largest dataset, we identified ten isolates with homozygous variants: five carried the 361 E/K known RNAi-deficient variant, one was homozygous for 365 I/V, one was homozygous for 341 R/K, and three were homozygous for 346 T/N. The known 361 E/K isolates are all closely related in cluster 1, while the other homozygous variants are at disparate places in the phylogeny. Their limited spread implies a potential fitness cost or selective constraint associated with loss of RNAi function. As our study was predominantly *in* *silico*, we did not assess the potential biological impact of these variants. In the future, it will be important to explore the differential functional impacts of the various variants to better understand their roles in *C. albicans* biology.

This study refines the phylogenetic structure of *C. albicans* using phased whole-genome data, resolving both known and novel clusters with greater clarity and proposing standardized definitions to improve cross-study comparisons. While clade distribution appears to show some structure by isolation source, our results suggest this pattern is largely confounded by geography. The predominance of the a/α *MTL* configuration supports largely clonal reproduction, while the presence of *MTL* homozygotes and a moderately high level of admixture indicates a potential for recombination. Aneuploidy and CNVs lack consistent geographic or ecological patterns, suggesting they may reflect transient responses to environmental stress. Although environmental isolates are underrepresented, their genetic overlap with human-associated strains and broader clade diversity (particularly in Europe) point to the environment as a potential reservoir and challenge models of strictly vertical transmission. Lastly, the discovery of novel mutations in RNAi components, including Ago1, highlights the need for functional studies to understand their impact on gene regulation and host interactions. Together, these findings provide a framework for *C. albicans* phylogenetics for exploring the evolutionary and ecological dynamics of *C. albicans*.

## Methods

### Acquisition of sequence data

The NCBI SRA database was searched in July 2024 for all deposited Illumina sequenced *C. albicans* whole-genome sequences^44^. Isolates from experimental evolution studies were excluded. Additionally, only samples with ≥80% of reads mapping to the reference genome, breadth of coverage ≥80%, and mean depth of coverage ≥30× were considered (following human WGS analyses^60,61^). In total, 1088 isolates from 22 publications were retained^10,14,25,42,45–48,20,59,62–73^. In addition, we included fastq data generated by the Joint Genome Institute from seven newly sequenced environmental isolates (four from soil, one from duff, and two from fruit) and 83 newly sequenced isolates acquired in 2012 and 2018 from the microbiology lab of the major hospital in Manitoba (Table S1) for a total of 1178 isolates (“complete isolate set”). The isolate collection included 361 isolates from 95 individuals with more than one isolate (“intrapopulation isolates”): 71 people with isolates taken from multiple time points, 24 people with multiple isolates taken simultaneously from the same time point (either at the same or different body sites) (Table S1). When necessary to avoid potential bias, when intrapopulation isolates from the same body site clustered monophyletically, we randomly selected a single isolate from each monophyletic group for each individual.

### DNA extraction and sequencing of Manitoba isolates

Genomic DNA was extracted from single colonies of 83 Manitoba isolates using a standard phenol-chloroform protocol previously described^74^. DNA quality and concentration were assessed using the Thermo Scientific™ NanoDrop 2000 and Qubit® 2.0 Fluorometer (with the Invitrogen™ Qubit™ dsDNA BR Assay Kit), respectively. The genomes of the 43 isolates from 2012 were sequenced by the Microbial Genome Sequencing Center (Pittsburgh, USA) using the Illumina NextSeq 550 sequencing technology with paired-end reads of 150 bp. The bcl-convert v3.9.3 package (https://support-docs.illumina.com/SW/BCL_Convert/Content/SW/FrontPages/BCL_Convert.htm) was used in demultiplexing, quality control, and adapter trimming. The genomes of 40 isolates from 2018 were sequenced by the Genome Quebec Innovation Center in Montreal using NovaSeq6000 S4 sequencing technology with paired-end reads of 150 bp. Reads have been deposited at the National Center for Biotechnology Information (NCBI) Sequence Read Archive under BioProject ID PRJNA991137.

### Read mapping and variant calling

Reads were inspected with FastQC v0.11.5 (https://www.bioinformatics.babraham.ac.uk/projects/fastqc/) and trimmed and filtered with Trimmomatic v0.36^75^. Read mapping and variant calling were performed using HaploTypo v1.0.1^76^ using the default parameters. Briefly, Haplotypo is run in four successive modules (see scripts in https://github.com/Gabaldonlab/haplotypo:) read mapping (mapping.py), variant calling (var_calling.py), inference of true alternative variants for each haplotype (VCFcorr_alleles.py), and reconstruction of phased haplotypes (haplomaker.py). Filtered reads were mapped with readgroups added onto the phased haplotypes (hapA and hapB) of the A22 SC5314 reference genome^16^ with BWA-MEM v0.7.18^77^. Picard v3.1.0 (http://broadinstitute.github.io/picard) was used to sort the alignment, convert the SAM alignment to BAM format, and mark duplicate reads. Alignment quality was assessed with CollectAlignmentSummaryMetrics from picard v3.1.0 (http://broadinstitute.github.io/picard) and consolidated across all samples with MultiQC^78^. Average depth of coverage was assessed with samtools coverage^79^ of samtools v1.20. Bcftools v1.19^79^ was used for calling and filtering variants using the default parameters in Haplotypo. The VCFcorr_alleles.py script of Haplotypo was used to compare the variant calling results from each sample against each of the two phased reference haplotypes to generate two VCF files, one for each haplotype, reporting the variants specifically observed in each of them while ignoring ambiguous genotypes (-amb 0).

### Comparison of trees constructed with and without haplotype information

To determine if considering haplotype information affected tree topology, we constructed a phylogeny using the 148 isolates from Ropars et al.^20^ (i.e., the existing WGS tree), as there exists cluster information for these isolates. We also retained one of the 35 cluster 13 isolates to root the phylogenies. We generated a phylogeny from the hapA (the main haplotype used in prior *C. albicans* phylogeny analyses) intermediate vcf files from running haplotypo. We also generated a phylogeny from the MLST alleles (*AAT1a*, *ACC1*, *ADP1*, *MPIb*, *SYA1*, *VPS13*, and *ZWF1b*) with loci extracted from the same intermediate vcf files. We then generated a phylogeny from the final consensus fasta files generated from considering haplotype information as detailed above.

To compare the trees, the pairwise distance between tree topologies was assessed using RF.dist and KF.dist functions from the phangorn package^80^ in the R Programming language. The normalized Robinson-Foulds (nRF) distance and branch score distance (KF) were calculated; nRF distance reflects the number of bipartitions differing between topologies, whereas the KF distance quantifies the difference in branch lengths and tree topology between the trees (01_Tree_topology_comparison.R). Therefore, two identical topologies will receive a value equal to 0 with both metrics. Conversely, distance values will increase (to max to 1 in nRF) as the compared trees become more different.

### WGS phylogeny construction from the complete isolate set

A new phylogeny was generated from 945 of “phylogenetically informative” isolates. This included 128 of the 361 intrapopulation isolates (as described above). A FASTA file for the reconstructed haplotype from each isolate was generated using the haplomaker.py of haplotypo script. This process ensures that heterozygous positions are not disregarded or replaced by IUPAC ambiguity codes, as is observed in many pipelines^81^. The two fasta sequences for each chromosome from each isolate sample were concatenated into a single sequence, and then all isolate sequences were combined into a single multiple sequence alignment file. This file was input to FastTree (2.1.11)^82^ in the double precision mode to construct a maximum-likelihood phylogenetic tree using the general time reversible model and the -gamma option to rescale the branch lengths. FastTree has been found to produce equally accurate trees with large datasets as other ML-based phylogeny predictors such as RAxML^83^, within a significantly shorter time^84^. The 37 *C. africana* (“cluster 13”) isolates were used to root the tree^85,86^. The resulting phylogeny was visualized and annotated with the Interactive Tree Of Life (iTOL, v5)^87^.

### MLST phylogenetic tree construction

Multilocus sequence typing (MLST) alleles were derived both from whole-genome sequencing (WGS) data and from publicly available MLST records. Extraction of MLST loci from WGS assemblies is not trivial, as allele boundaries can vary between isolates and may not be directly retrievable from draft genomes. To accurately recover MLST loci from WGS data, individual locus reference sequences were first aligned to the SC5314 A22 genome^16^ assembly to identify the precise genomic coordinates of each locus in each isolate and extract the corresponding sequences. Extracted loci were subsequently concatenated in a consistent order to generate full MLST profiles for each isolate. In addition, MLST sequences were downloaded from the PubMLST database to supplement the dataset. Duplicate isolates were identified and removed prior to phylogenetic analysis to avoid redundancy and over-representation of closely related samples. Concatenated MLST sequences were used to construct a phylogenetic tree representing the complete MLST dataset. The resulting tree was examined to confirm that distinct DSTs formed separate clades, verifying that sequence types were phylogenetically distinguishable within the dataset. The sequence types of the 83 newly sequenced Canadian isolates, as well as the seven environmental isolates from the USA, were determined using stringMLST^88^ with default parameters. The *Candida albicans* MLST database was downloaded from PubMLST. Comparison with the PubMLST database showed that only three of the 83 Canadian isolates, as well as the seven environmental isolates from the USA, corresponded to previously described DSTs; the remaining isolates did not match any known DSTs.

### Genetic cluster delineation

To delineate the clusters within the phylogeny, we ran TreeCluster^89^. TreeCluster uses several functions to agnostically identify clusters within phylogenetic trees. We selected methods that are optimized to identify clusters within the phylogeny (i.e., methods that have the “clade” suffix). This included max clade, which is the default method. We additionally examined the single linkage method as a representation of the three single linkage methods (single linkage cut, single linkage union). To identify a statistically well-supported phylogeny, we ran each method with threshold values from *t* = 0–1, increasing *t* by 0.001, for a total of 1000 values per method. We identified regions of parameter space where a range of threshold values yielded the same number of genetic clusters. We then visually inspected each genetic cluster assignment on the phylogeny using iTOL and compared it to the existing WGS tree.

### Heterozygosity analyses

Sites with missing data are common in short-read WGS datasets and can substantially bias estimates of heterozygosity. We therefore excluded isolates with more than 15% missing genotype positions from the heterozygosity analyses, retaining 745 isolates from the phylogenetically informative isolate set. This filtering step was applied specifically to heterozygosity calculations because missing data disproportionately inflate or deflate per-isolate heterozygosity estimates, whereas phylogenetic inference is comparatively robust to moderate levels of missing data due to the large number of informative sites contributing to tree topology. For this reason, isolates exceeding the missingness threshold were retained in phylogenetic analyses but excluded from heterozygosity comparisons. Average heterozygosities were calculated and statistically compared by isolation site and cluster after verifying the homogeneity of variances using Levene’s test.

Genome-wide heterozygosity at each was calculated using PLINK v2.0^90^. Bcftools was used to call variants in consensus mode to ensure that all sites were considered using the A22 hapA reference^16^. Genotype counts for each isolate were obtained by extracting the number of homozygous reference (hom), homozygous alternate (homalt), and heterozygous (het) genotypes from the VCF files. To calculate the genome-wide heterozygosity across the isolates, *bcftools query* (options -H and -f) was used to generate a table of genotypes in all positions of 738 isolates. A custom script (06-heterozygosity_analyses.R) was used to calculate and visualize the heterozygosity for 5 kb sliding window across the genome. Centromeric and subtelomeric regions (defined as 15 kb from the start and end of each chromosome) were excluded as they are known to be prone to artifactual errors in short-read data due to repeats.

### NGSAdmix

Admixture was estimated among isolates of the phylogenetically informative set. Genotype likelihoods were estimated directly from aligned sequencing reads using ANGSD^91^. Major and minor alleles were inferred from the data, and SNPs were identified using a likelihood ratio test with a significance threshold of *p* < 1 × 10⁻⁶. Minor allele frequencies were estimated for all retained sites. Genotype likelihoods were output in BEAGLE format for downstream population genetic analyses. All analyses were parallelized using 10 computational threads. Population structure was inferred using NGSadmix, which estimates individual ancestry proportions from genotype likelihoods. Analyses were performed using genotype likelihoods in BEAGLE format. We evaluated a range of ancestral population numbers (k = 6–20), running each value of k independently. Loci with a minor allele frequency below 0.05 were excluded. Each NGSadmix run was executed using 32 threads, and analyses were parallelized across K values using GNU Parallel. Admixture proportions were visualized in R.

### Aneuploidy analyses

We quantified aneuploidy and copy number variation (CNV) (duplications or deletions of smaller genomic segments) in the complete genome isolate set. Sequencing reads were aligned to the reference genome using BWA-MEM v0.7.18 as detailed above. Post-alignment, we used samtools depth to calculate the depth of coverage at each genomic position from each BAM file, generating a comprehensive coverage profile across all chromosomes. Coverage data was processed and visualized using a custom R script (05a-Aneuploidy.R). The script calculates the average read depth within non-overlapping 5-kb bins across the genome. The median number of reads per chromosome is calculated and used to normalize read depth across bins. Chromosomal aneuploidies and small regions of elevated copy number (CNVs) were visually identified by two people independently, based on the generated plots. Where coverage across at least one chromosome (or chromosome part) was a non-integer number, read depth was recalculated to have a base ploidy of triploid or tetraploid and again scored visually. CNV breakpoints were manually determined by determining the bin where read depth changed, which is typically very clear. Example graphs used for quantification are provided in Fig. S13.

### *MTL* analyses

The mating type-like (*MTL*) locus in the 945 isolates was identified by aligning sequencing reads to both haplotypes of chromosome 5 from the A22 reference genome (A22-s08-m01-r09 from the Candida Genome Database) using bcftools. Samtools depth was employed to calculate both the depth and breadth of coverage across the **MTLa** region (Ca22chr5A_C_albicans_SC5314:393493–394455 and 394560–395220) and the **MTLα** region (Ca22chr5B_C_albicans_SC5314:395642–396223 and 401608–402227). Loci with coverage <0.5 were considered inactive or absent.

### Identifying variants in the *AGO1* gene

We sought to quantify the prevalence of mutations in the Ago1 PAZ domain, linked to RNAi activity, in the 908 isolate set. A BLAST search of the gene was used to pinpoint the exact coordinates of the PAZ domain in the A22 *C. albicans* chromosome 4 reference (i.e., chr4_A:1408039-1408347). The corresponding region was then extracted from the vcf files from all isolates using *bcftools filter* and converted to a multiple sequence alignment file. Missing data were assumed to be homozygous consensus. For heterozygous regions, the homozygous and alternate alleles were independently incorporated into the reference to construct the haplotype sequences for each isolate to identify unique PAZ domain sequences. The nucleotide sequences were aligned using Clustal Omega as implemented in SnapGene. The aligned sequences were subsequently translated into amino acid sequences using amino acid codon table 12 (“alternative yeast,” from https://www.kazusa.or.jp/codon/). The frequencies of unique PAZ domain sequences were then calculated, and the regions of difference were identified.

## Supplementary information

Supplementary Information

TableS1

TableS2

TableS3

TableS4

## Data Availability

FASTQ files generated for this project have been deposited at the National Center for Biotechnology Information (NCBI) Sequence Read Archive under BioProject ID PRJNA1418157. All data and code required to reproduce all statistical analyses and visualizations (excepting the phylogenies) are available at https://github.com/microstatslab/Calbians_phylogenetics. Due to file size limitations (>50 MB), some large files could not be deposited in the repository and are available from the authors upon reasonable request.
