# Supplementary material for: Linking geography, isolation source, and genomic diversity in a global Candida albicans phylogeny
Source: Npj Fungal Sci. 2026 Nov 5;1(1):7. doi: 10.1038/s44512-026-00003-2 (PMC13739746; doi:10.1038/s44512-026-00003-2)
Supplement: Supplementary file 1 [file 44512_2026_3_MOESM1_ESM.pdf]

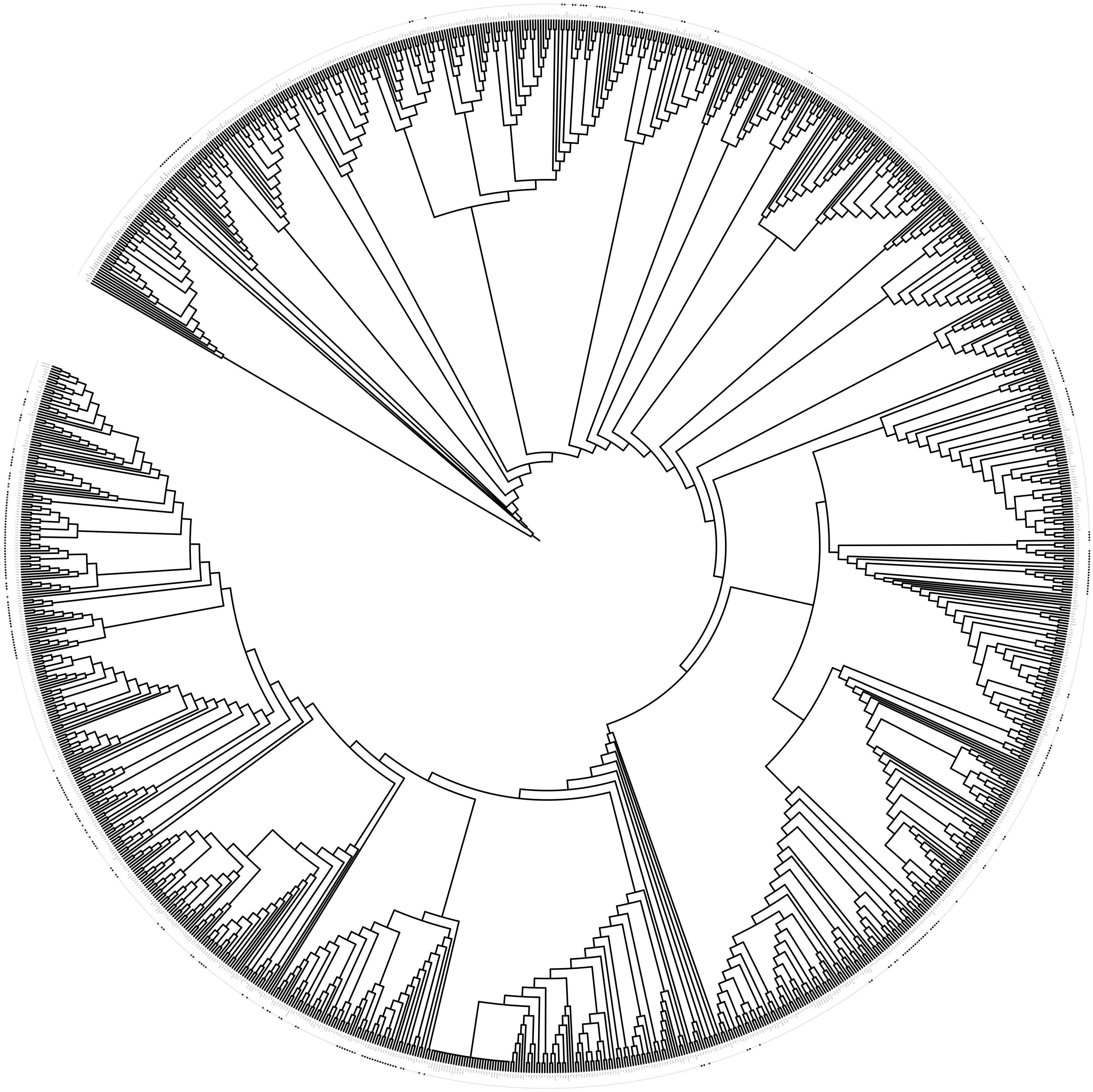

Figure S1. Intra-population strain set. Black dots indicate isolates that were removed for the phylogenetically informative set. This figure does not include all, but only the retained isolate from the three participants that were published in Adamu-Bukari 2025.

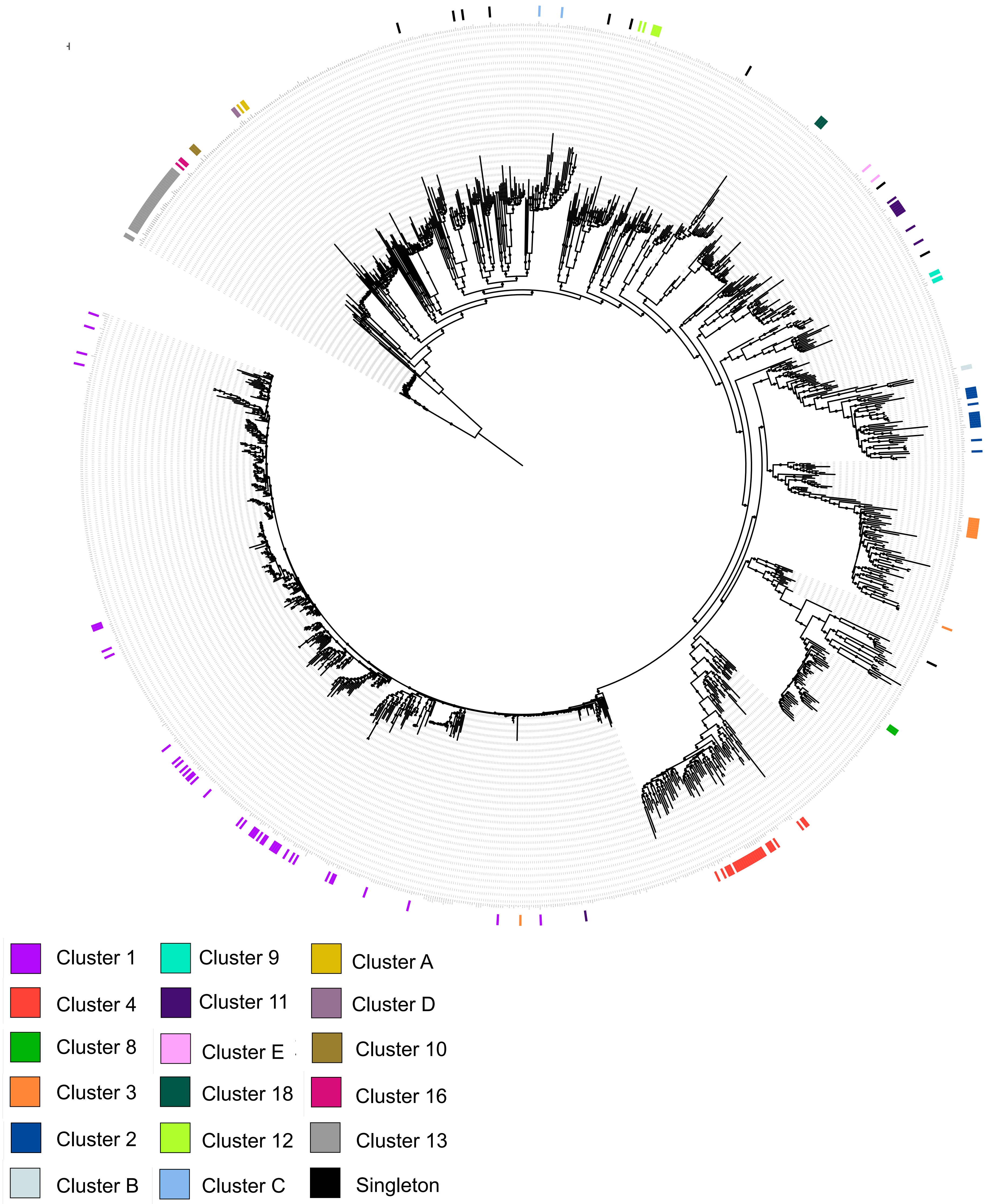

Figure S2. Phylogeny depicting the positions of isolates in the existing WGS tree from Ropars et al 2018 in an outer ring. The legend is arranged in anti-clockwise order of the isolates predicted clusters, the cluster designation from Ropars et al 2018 are in the outer ring.

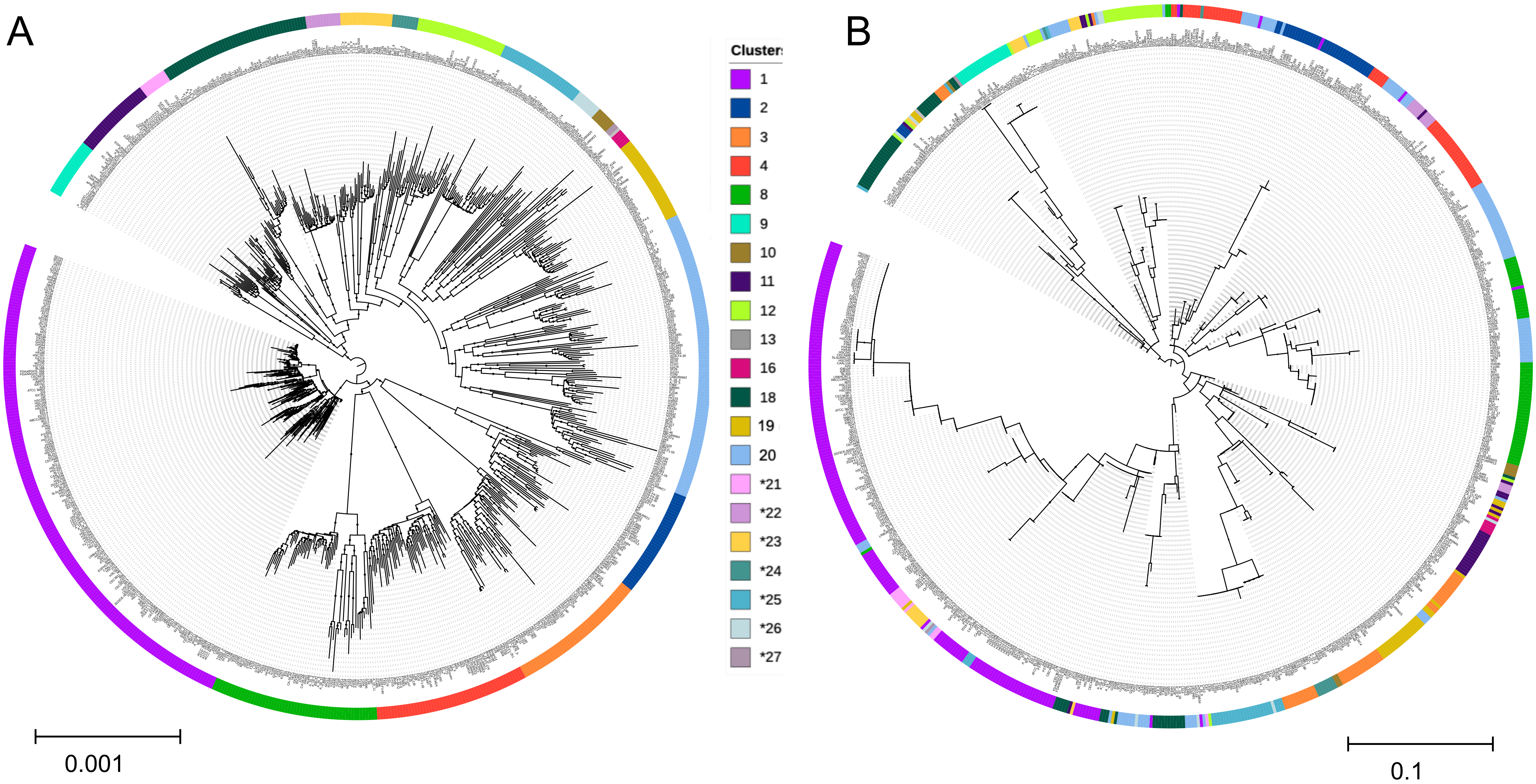

Figure S3. Comparison of phylogenies of *Candida albicans* isolates. A) The phylgoeny generated from short-read WGS sequences compared to B) the phylogney generated from concatenated MLST sequences. Both trees are rooted at midpoint. The outer ring colours indicates genetic clusters defined from the WGS tree.

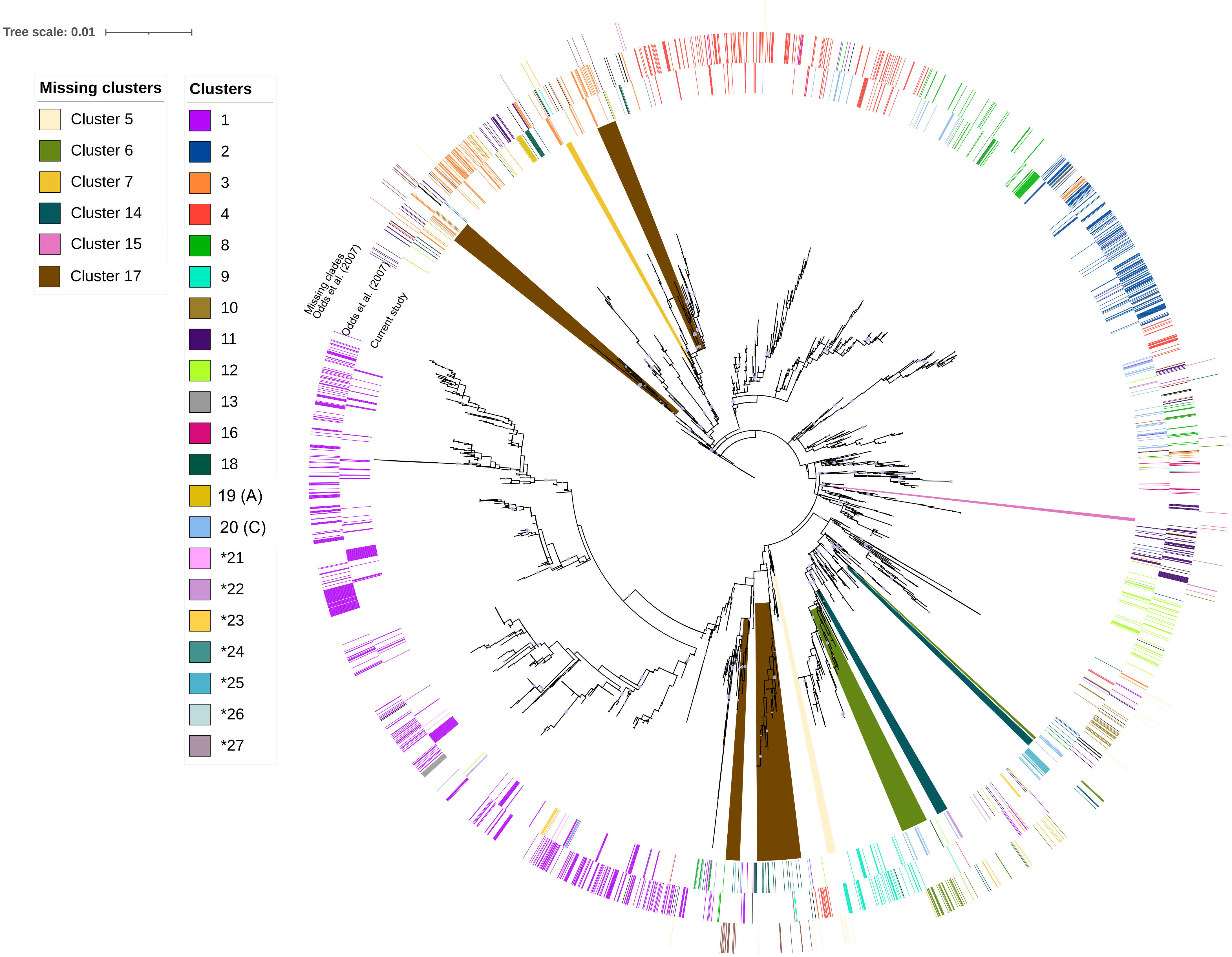

Figure S4: Isolates from the missing Odds et al. (2007) clusters do not correspond well to existing or new WGS cluster assignments.

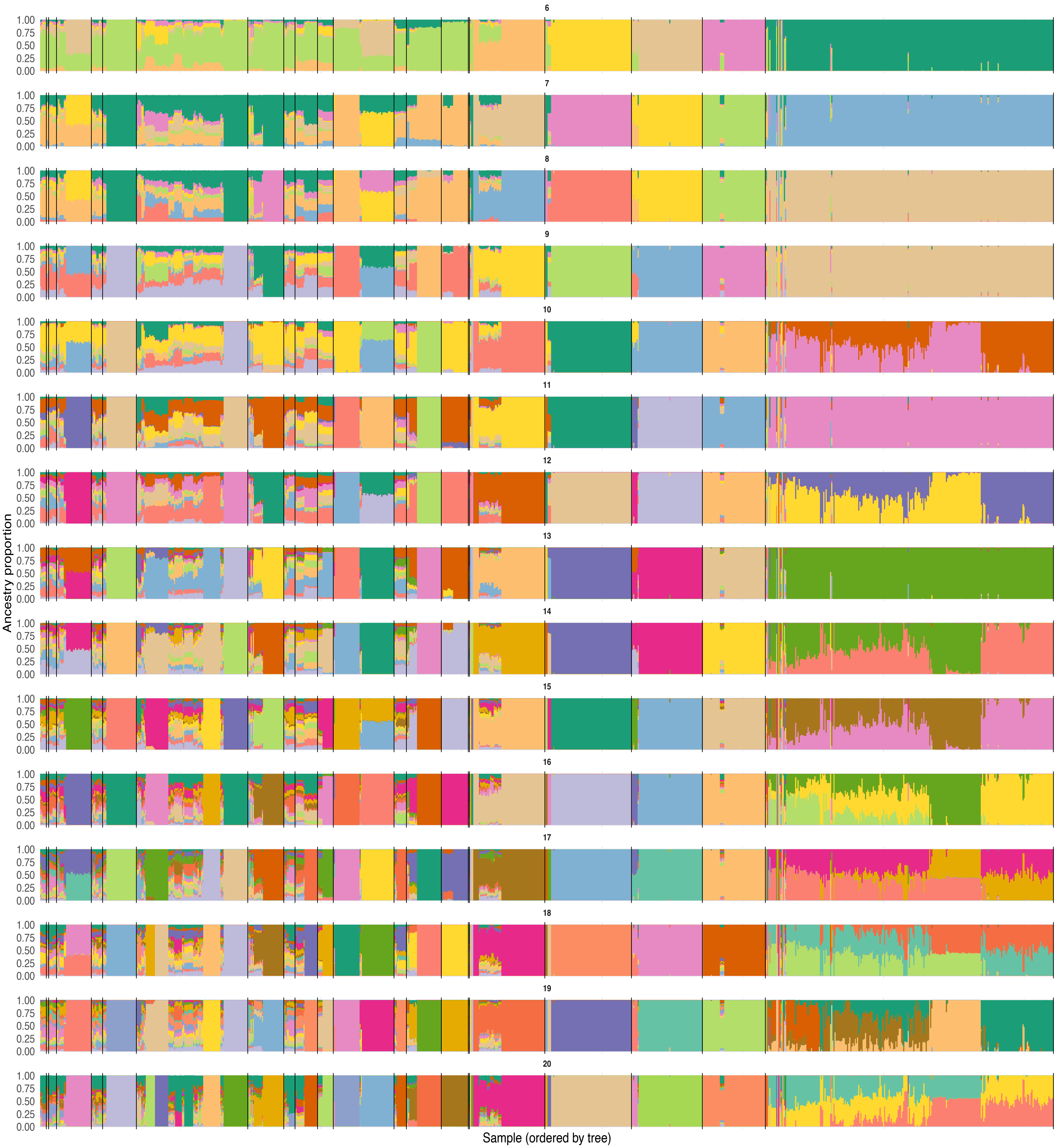

Figure S5. Admixture analysis from k = 6 to k = 20. A) Each vertical bar represents an individual isolate, ordered according to its position in the phylogenetic tree, and colored segments indicate the proportion of ancestry assigned to each inferred cluster. Vertical black lines denote boundaries between proposed phylogenetic clades.

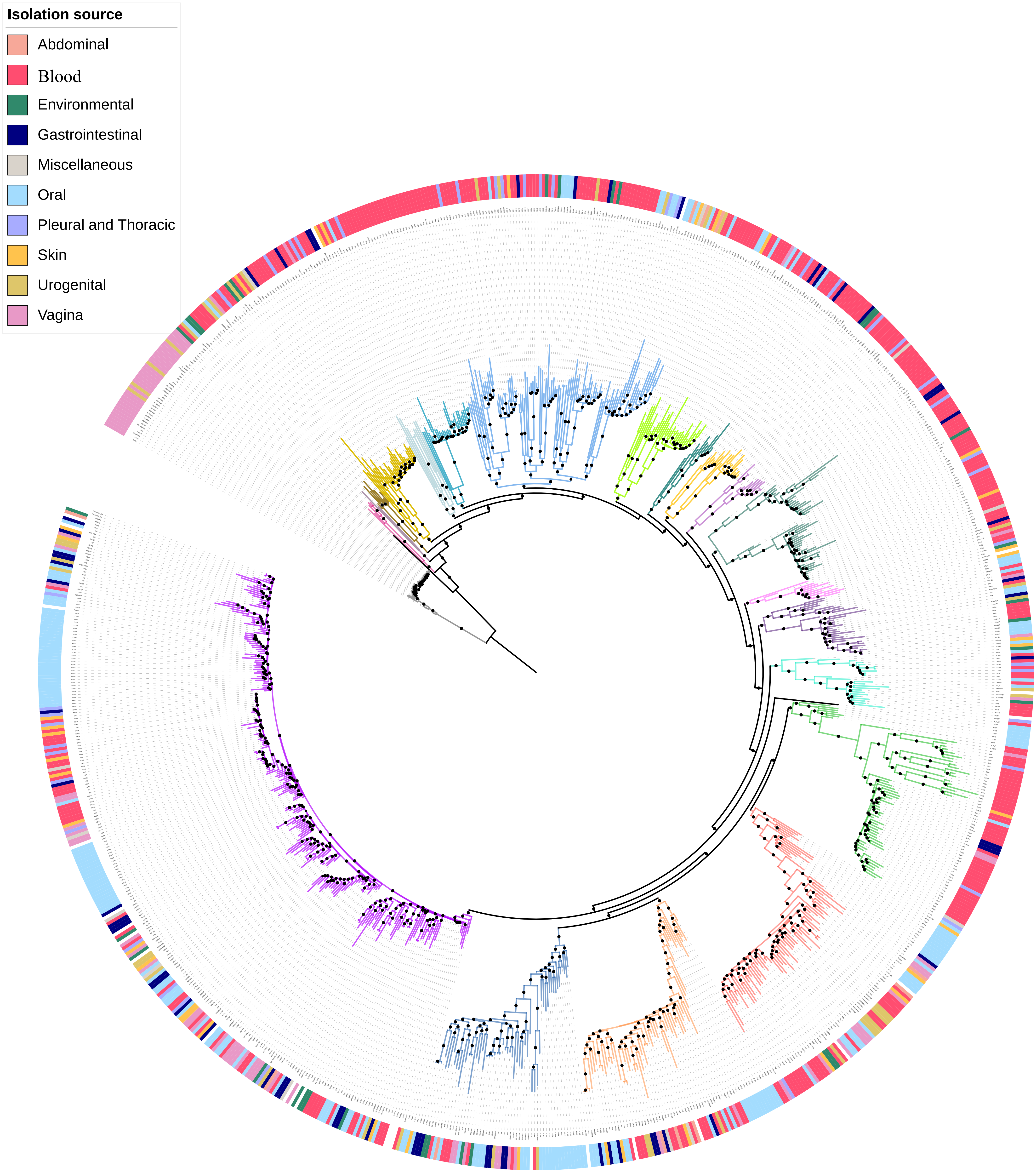

Figure S6. Phylogeny of isolates by source of isolation. Maximum likelihood phylogeny of all isolates, with branches colored by clade to indicate genetic relationships. The outer ring represents the source of isolation, with colors corresponding to different source.

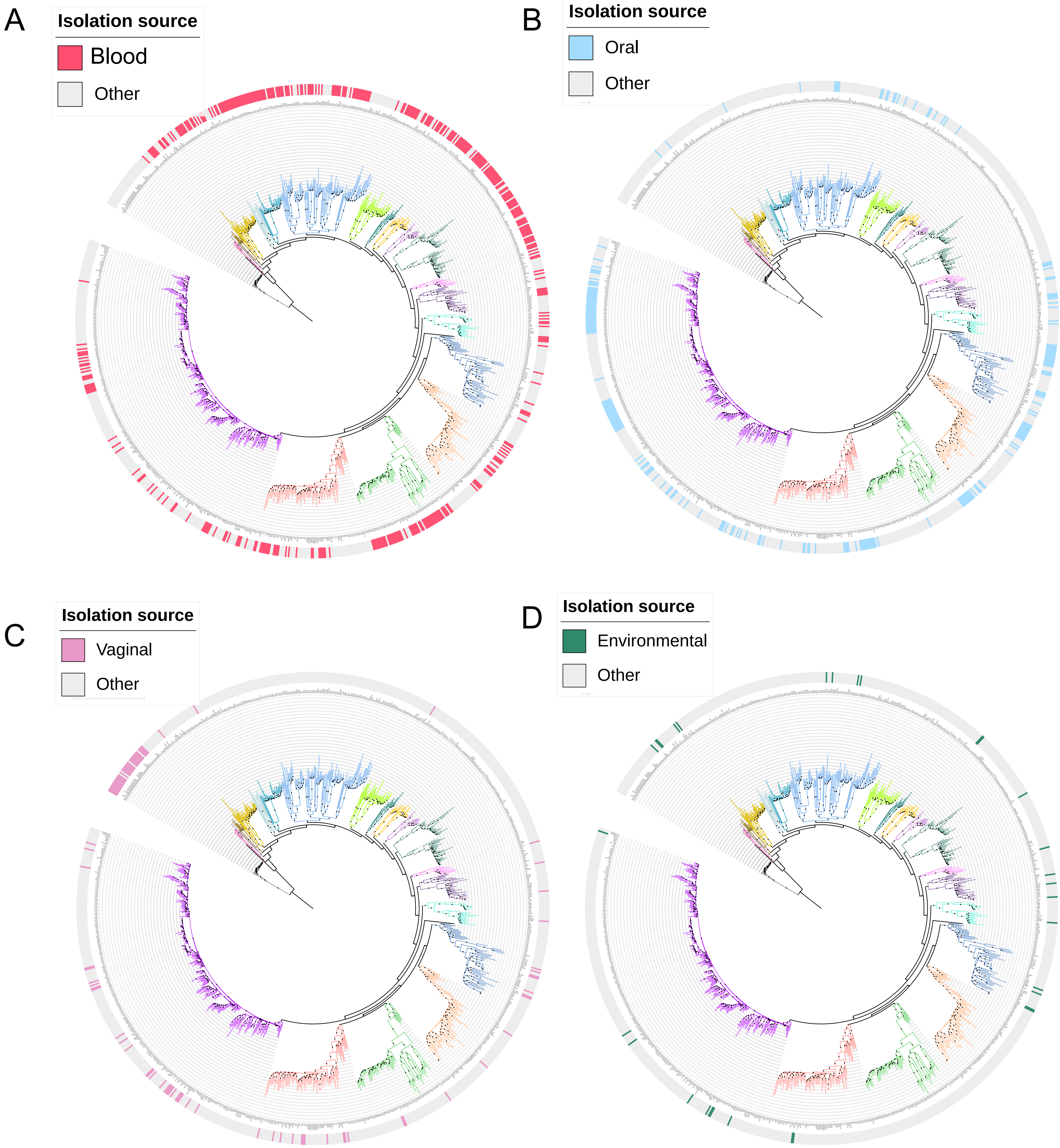

Figure S7: Maximum-likelihood phylogenies depicting the distribution of the *C. albicans* isolates by the source of isolation (A, blood; B, oral; C, vaginal; D, environmental). Branches are colored according to the genetic clusters to which the isolates belong. Black circles on the branches indicate bootstrap support values greater than 0.9. The outer ring represents the source of isolation, with colors corresponding to different sources. Four main isolation sources have been shown due to preponderance and/interest.

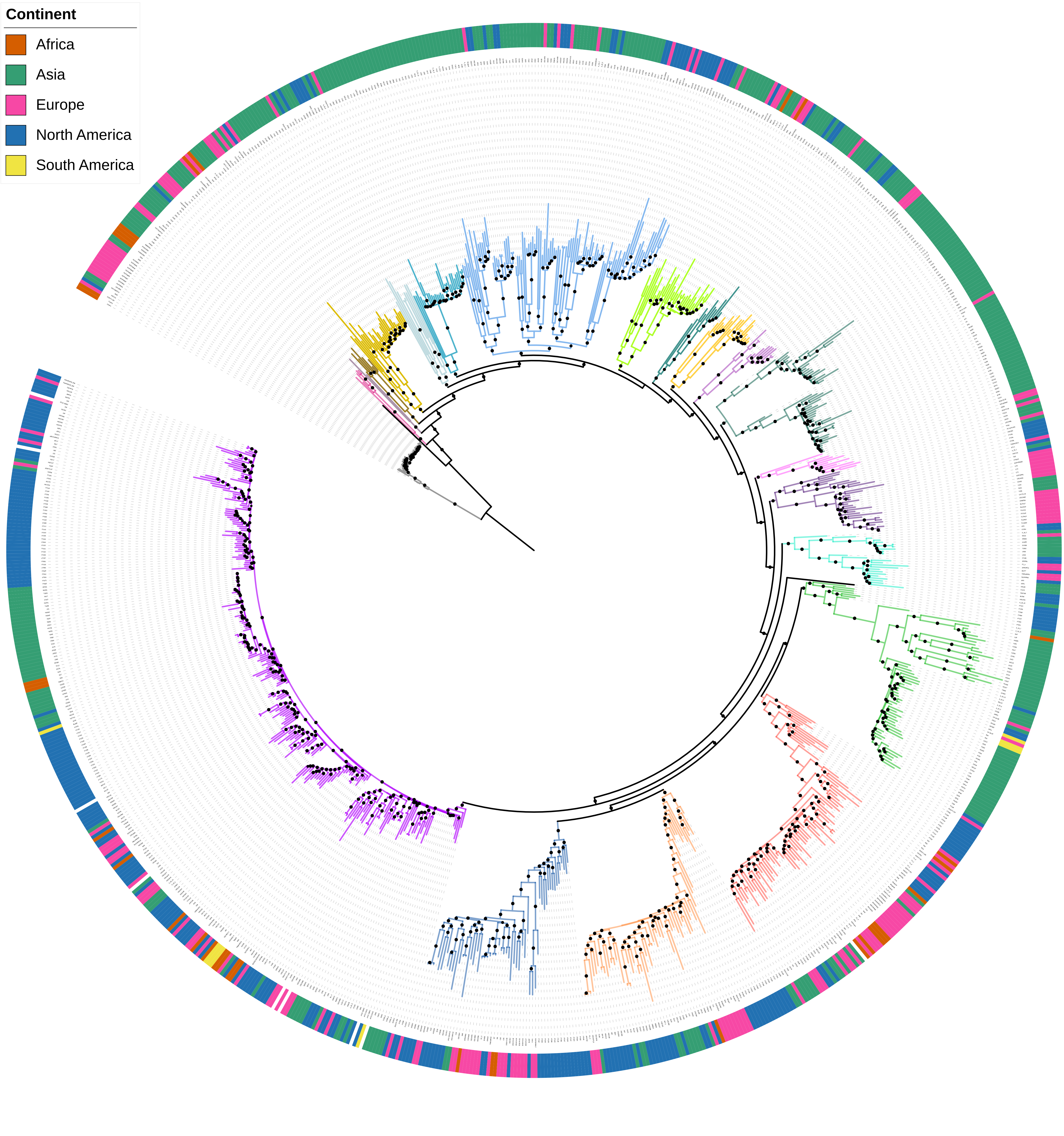

Figure S8. Phylogeny of isolates by geography. Maximum likelihood phylogeny of all isolates, with branches colored by clade to indicate genetic relationships. The outer ring represents the geographical source of isolation, with colors corresponding to different continent.

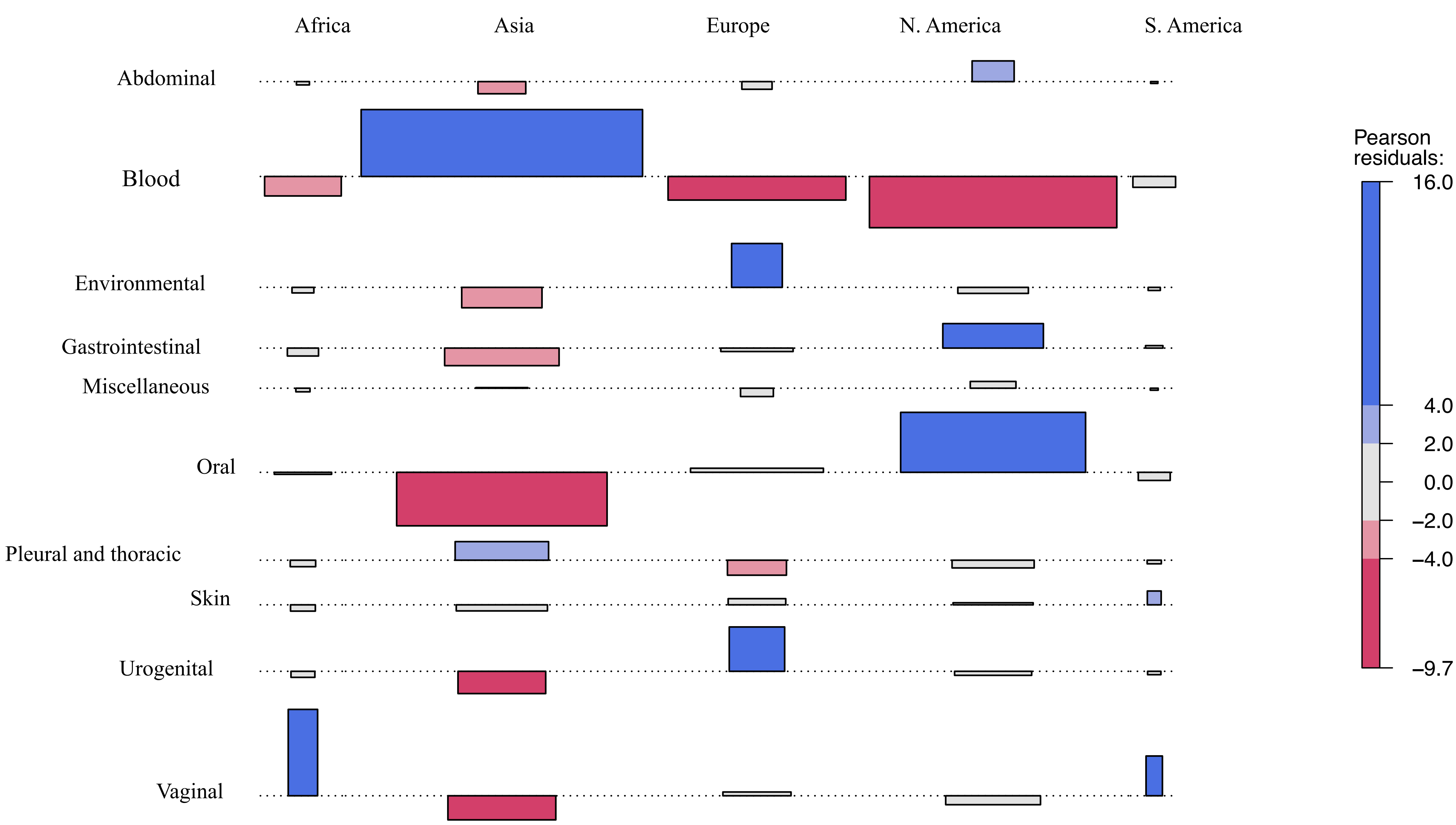

Figure S9. Geographic structure in isolation source and clade distribution. Association plot showing deviations from independence between isolation source and continent. Each bar represents a cell in the contingency table, with height and color indicating the direction and strength of standardized residuals. Blue bars represent combinations that occur more frequently than expected under independence; red bars indicate underrepresentation. The strong association (χ^2 $p < 0.00001$, Cramér's $V = 0.55$) indicates that isolation source is strongly nested within geography, reflecting structured sampling or ecological differences across regions.

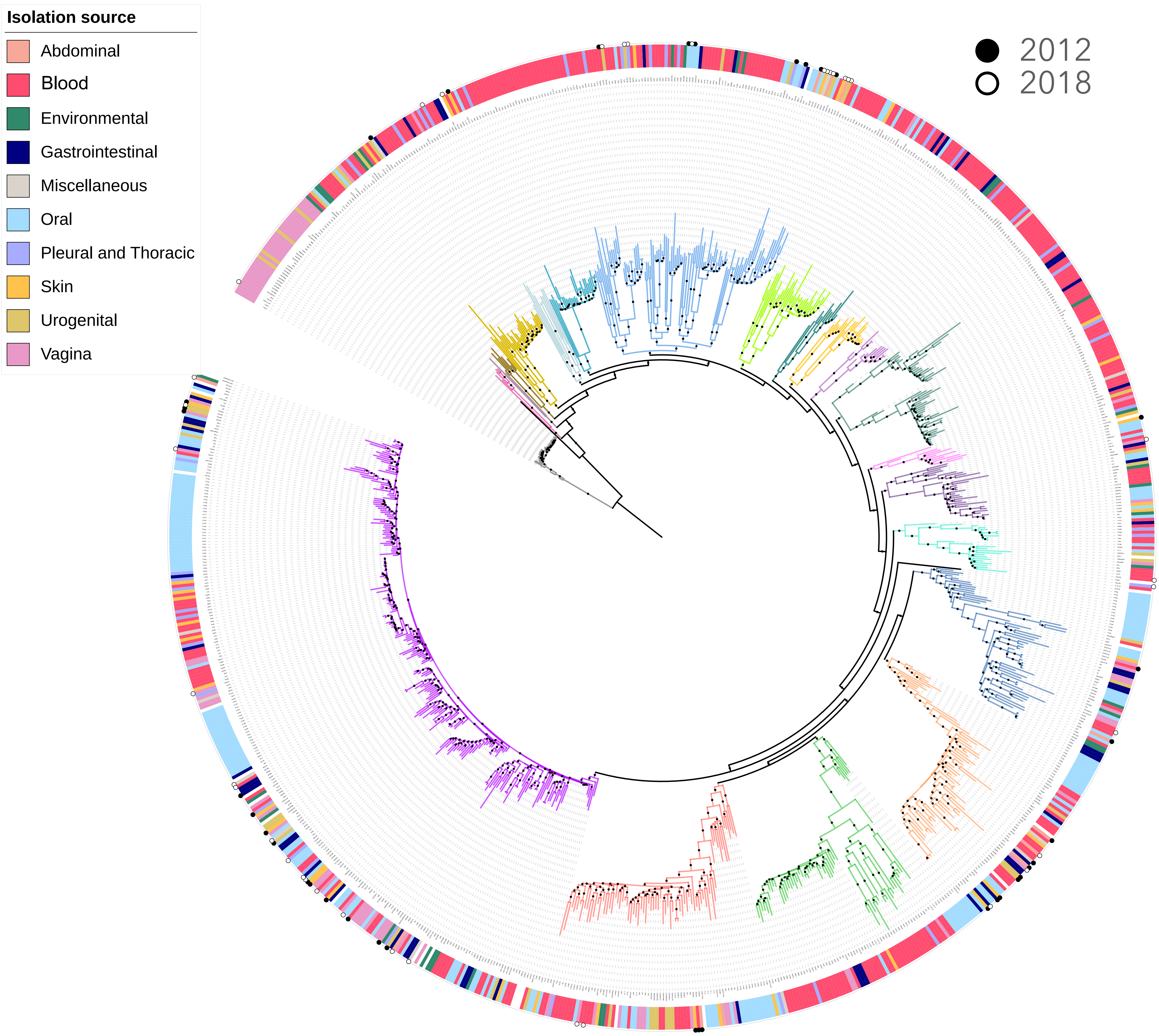

Figure S10. Phylogenetic placement of Manitoba isolates. Maximum likelihood phylogeny of all isolates, with branches coloured by clade to indicate genetic relationships. The inner ring shows the source of isolation, while the outer dots mark the Manitoba isolates included in this study. Open dots represent isolates collected in 2018, and closed dots represent isolates collected in 2012.

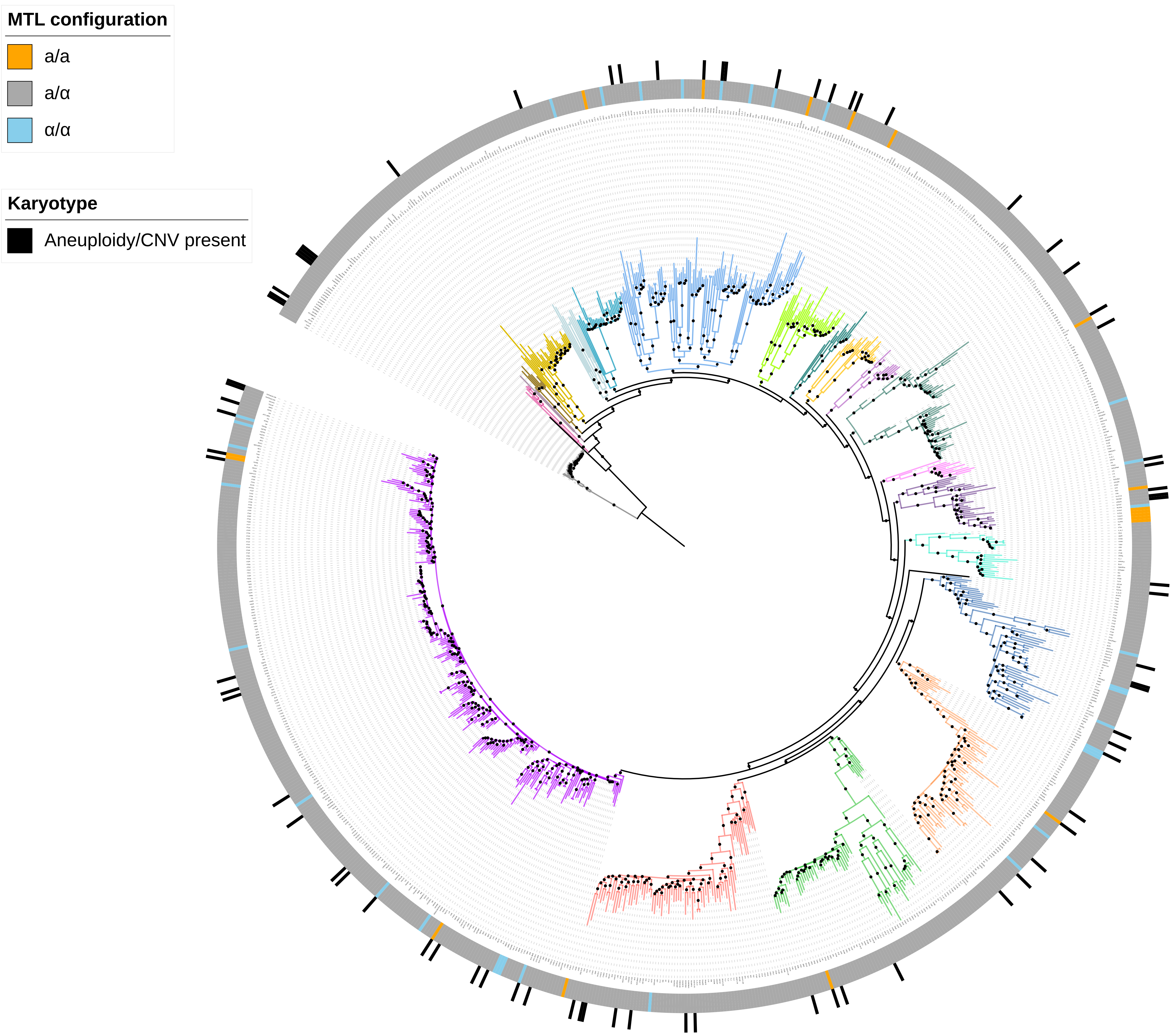

Figure S11. Distribution of aneuploidies, CNVs, and MTL locus genotype across the phylogeny. Maximum likelihood phylogeny of all isolates, with two concentric annotation rings. The outer ring indicates isolates exhibiting aneuploidies and/or copy number variations (CNVs), while the inner ring represents the distribution of different MTL locus configurations.

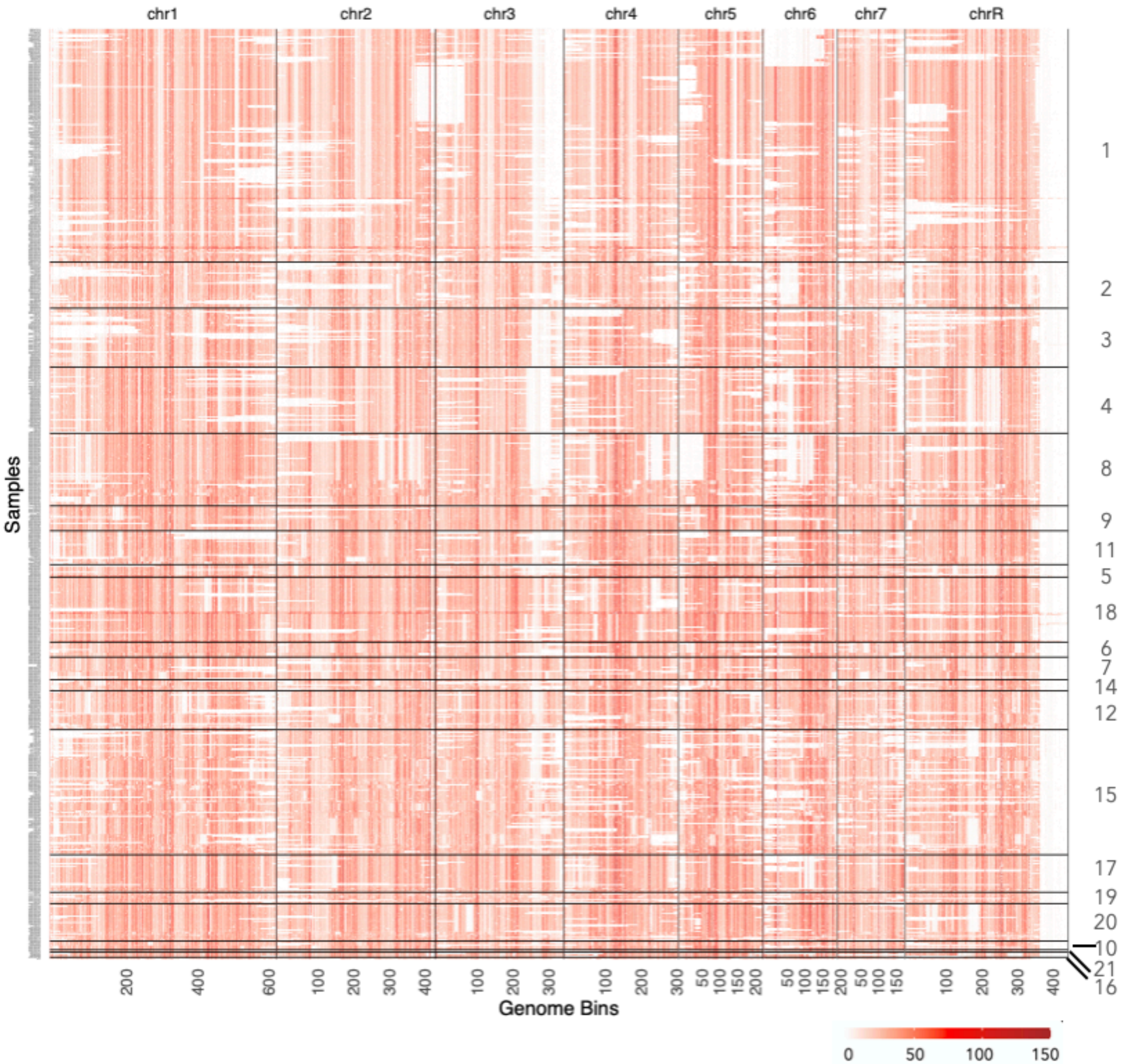

Figure S12: Density of heterozygous SNPs in 744 *C. albicans* isolates, in 5 kb windows. Each row represents an isolate. Isolates are ordered according to their order on the phylogeny. Thick vertical black lines delimit chromosomes (from 1 to 7 and R). Horizontal white stripes are indicative of recent LOH events. The scale bar represents density of heterozygous SNPs per 5 kb window, from a low density(white for 0) to a high density in dark red.

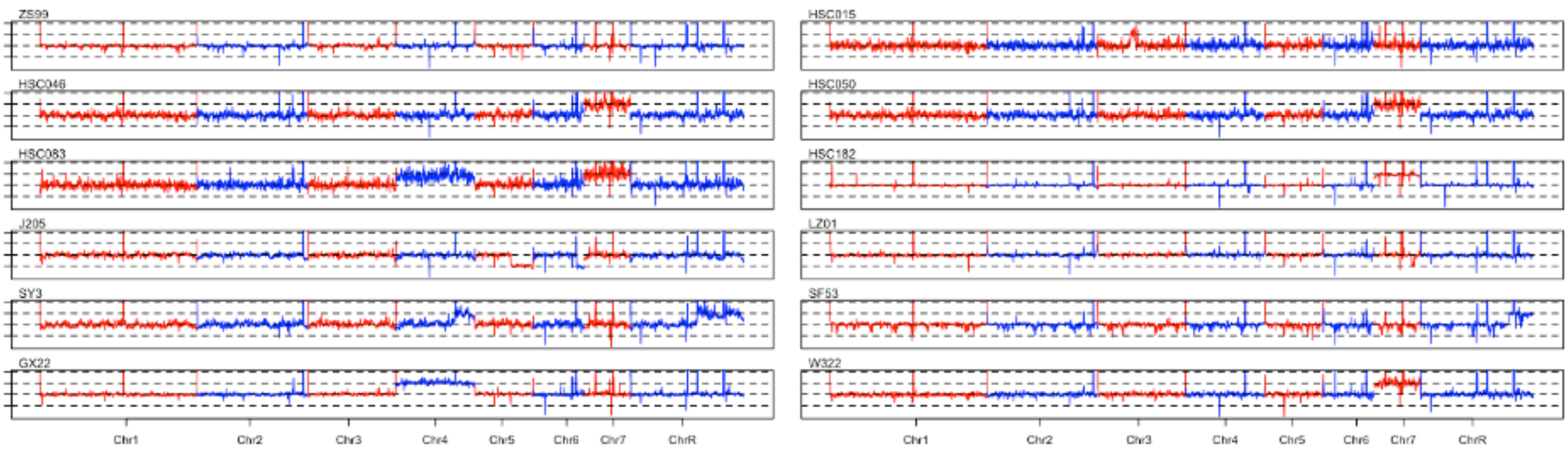

Figure S13: Example ploidy tracks used for quantification of aneuploidies in *C. albicans* generated with a R script. The alternating blue and red segments represent chromosomes normalized to a baseline ploidy of 2. Aneuploidies (e.g., trisomy of chromosome 7 in isolate HSC046) and copy number variations (e.g., on chromosome 3 in isolate HSC015) are shown as deviations from the baseline ploidy.
